# Bronchial epithelial DNA methyltransferase 3b dampens pulmonary immune responses during *Pseudomonas aeruginosa* infection

**DOI:** 10.1371/journal.ppat.1009491

**Published:** 2021-04-01

**Authors:** Wanhai Qin, Xanthe Brands, Cornelis van’t Veer, Alex F. de Vos, Jean-Claude Sirard, Joris J. T. H. Roelofs, Brendon P. Scicluna, Tom van der Poll

**Affiliations:** 1 Center of Experimental & Molecular Medicine, Amsterdam University Medical Centers, location Academic Medical Center, University of Amsterdam, Amsterdam, the Netherlands; 2 Université de Lille, CNRS, Inserm, CHU Lille, Institut Pasteur de Lille, Lille, France; 3 Department of Pathology, Amsterdam University Medical Centers, location Academic Medical Center, University of Amsterdam, Amsterdam, the Netherlands; 4 Department of Clinical Epidemiology, Biostatistics and Bioinformatics, Amsterdam University Medical Centers, location Academic Medical Center, University of Amsterdam, Amsterdam, the Netherlands; 5 Division of Infectious Diseases, Amsterdam University Medical Centers, location Academic Medical Center, University of Amsterdam, Amsterdam, the Netherlands; Universite de Reims Champagne-Ardenne, FRANCE

## Abstract

DNA methyltransferase (Dnmt)3b mediates *de novo* DNA methylation and modulation of Dnmt3b in respiratory epithelial cells has been shown to affect the expression of multiple genes. Respiratory epithelial cells provide a first line of defense against pulmonary pathogens and play a crucial role in the immune response during pneumonia caused by *Pseudomonas (P*.*) aeruginosa*, a gram-negative bacterium that expresses flagellin as an important virulence factor. We here sought to determine the role of Dntm3b in respiratory epithelial cells in immune responses elicited by *P*. *aeruginosa*. *DNMT3B* expression was reduced in human bronchial epithelial (BEAS-2B) cells as well as in primary human and mouse bronchial epithelial cells grown in air liquid interface upon exposure to *P*. *aeruginosa* (PAK). Dnmt3b deficient human bronchial epithelial (BEAS-2B) cells produced more CXCL1, CXCL8 and CCL20 than control cells when stimulated with PAK, flagellin-deficient PAK (PAKflic) or flagellin. Dnmt3b deficiency reduced DNA methylation at exon 1 of *CXCL1* and enhanced NF-ĸB p65 binding to the *CXCL1* promoter. Mice with bronchial epithelial Dntm3b deficiency showed increased *Cxcl1* mRNA expression in bronchial epithelium and CXCL1 protein release in the airways during pneumonia caused by PAK, which was associated with enhanced neutrophil recruitment and accelerated bacterial clearance; bronchial epithelial Dnmt3b deficiency did not modify responses during pneumonia caused by PAKflic or *Klebsiella pneumoniae* (an un-flagellated gram-negative bacterium). Dnmt3b deficiency in type II alveolar epithelial cells did not affect mouse pulmonary defense against PAK infection. These results suggest that bronchial epithelial Dnmt3b impairs host defense during *Pseudomonas* induced pneumonia, at least in part, by dampening mucosal responses to flagellin.

## Author summary

The respiratory epithelium provides a first line of defense against respiratory pathogens. *Pseudomonas (P*.*) aeruginosa* is a common causative pathogen in pneumonia and its important virulence factor flagellin is a potent activator of epithelial cells. DNA methyltransferase (Dnmt)3b is an enzyme that mediates *de novo* methylation of DNA. We here tested the hypothesis that Dnmt3b is involved in the immune response generated in airway epithelial cells upon infection with *P*. *aeruginosa*. Using a combination of *in vitro* investigations with human bronchial epithelial cells and *in vivo* airway infection models in mice with targeted deletions of the gene encoding Dnmt3b in specific subtypes of airway epithelial cells we demonstrate that Dnmt3b in bronchial but not type 2 alveolar epithelial cells impairs host defense during *Pseudomonas* induced pneumonia, at least in part by inhibiting mucosal responses to flagellin, by an effect on epithelial cell DNA methylation. We report a thus far unknown role for bronchial epithelial cell Dnmt3b in the innate mucosal immune response to a common respiratory pathogen, providing insight into the regulatory machinery involved in reprograming of epithelial cells during pneumonia.

## Introduction

*Pseudomonas (P*.*) aeruginosa* is a gram-negative flagellated bacterial pathogen and a common cause of pneumonia in hospitalized patients and those who suffer from chronic lung diseases [1,2]. The emergence of multidrug resistant *Pseudomonas* strains is a major health care concern, with reported rates of 15–30% in some geographical areas [3].

The respiratory epithelium provides a first line of defense against respiratory pathogens by producing a physical barrier and by releasing antimicrobial peptides, as well as chemotactic mediators [4]. Lung epithelial cells can be activated through a variety of receptors that recognize pathogens or components thereof. *P*. *aeruginosa* expresses a flagellum, which is important for its motility and is a major determinant of pathogenicity. Flagellin is the structural component of flagella recognized by Toll-like receptor (TLR) 5, thereby playing a key role in the induction of an innate immune response to infection with *Pseudomonas* [5,6]. TLR5 is abundantly expressed on the respiratory epithelium and triggering of this receptor results in the activation of the common TLR adaptor myeloid differentiation factor (MyD) 88 and subsequently nuclear factor (NF)-κB [5,6]. We and others previously documented a role for MyD88—dependent signaling in respiratory epithelial cells in boosting host defense during *Pseudomonas* pneumonia in mice [7,8,9].

The extent of DNA methylation influences gene transcription and chromatin structure, and can change during bacterial infection [10,11]. DNA methyltransferase (Dnmt)3b is one of the main enzymes mediating *de novo* DNA methylation [12]. Recent studies have suggested that alterations in DNA methylation mediated by Dnmt3b activity may affect transcriptional regulation in a context and cell specific way. The airway epithelium expresses Dnmt3b, which is enhanced by exposure to cigarette smoke [13]. Modulation of airway epithelial cell Dnmt3b expression impacts the expression of multiple genes [14]. Furthermore, exome sequencing associated variants of *DNMT3B* with community-acquired *P*. *aeruginosa* infection in children [15]. Together these results led us to hypothesize that respiratory epithelial Dnmt3b might play a role in host defense against *P*. *aeruginosa* infection. To test this hypothesis we evaluated innate immune responses induced in airway epithelial cells with modified Dnmt3b expression *in vitro* and in mice with airway epithelial cell specific deletion of Dnmt3b *in vivo* upon exposure to wild-type *P*. *aeruginosa*, or isogenic flagellin deficient *P*. *aeruginosa*.

## Results

### Dnmt3b reduces *Pseudomonas aeruginosa* induced chemokine production by bronchial epithelial cells *in vitro*

**Fig 1:**
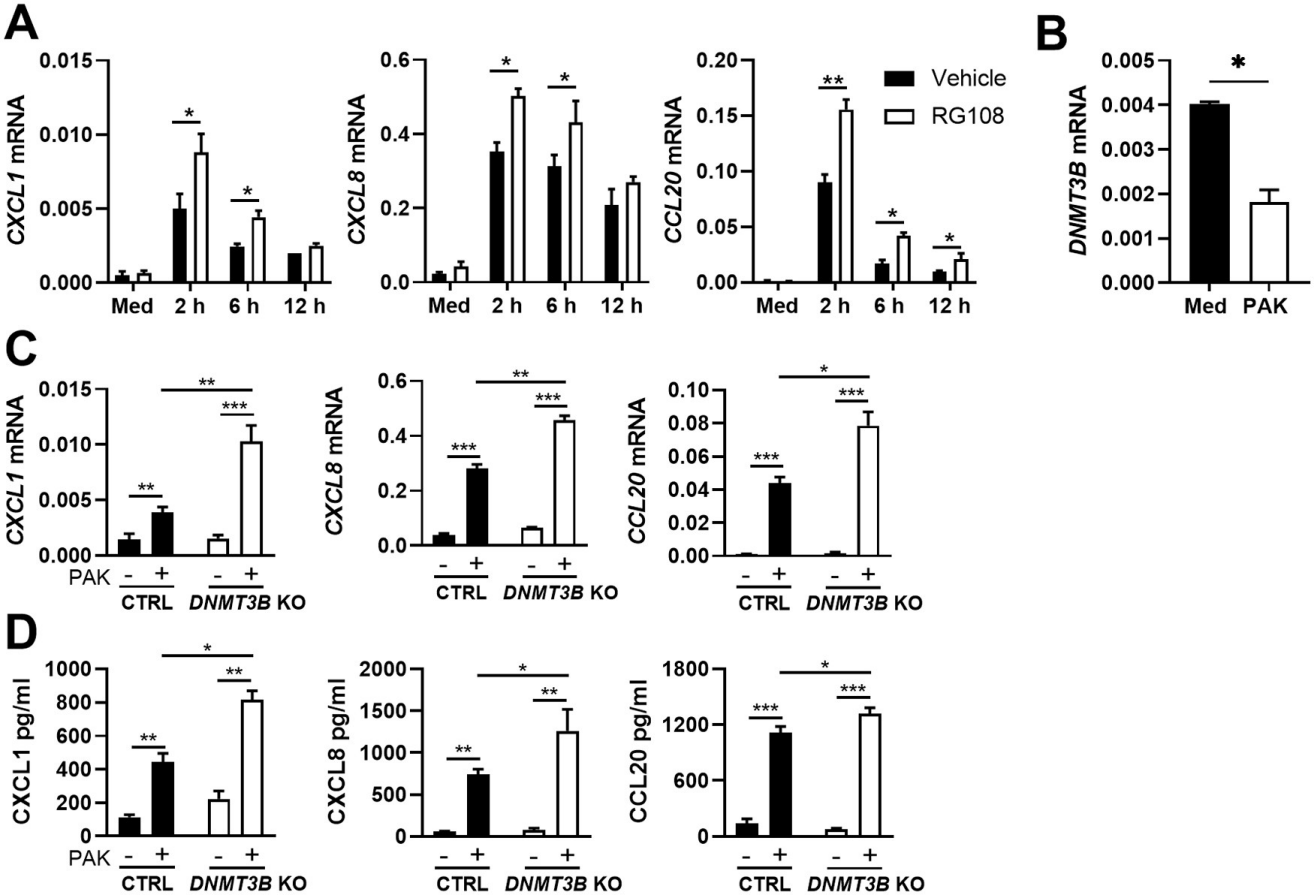
Dnmt3b inhibition in BEAS-2B bronchial epithelial cells promotes *Pseudomonas aeruginosa* induced chemokine production *in vitro*. BEAS-2B cells were pretreated with the DNMT inhibitor RG108 at 10 μM/ml (open bars) or vehicle (black bars) for 12 hours and then stimulated with heat killed PAK (MOI = 50) or medium control for 2, 6 or 12 hours. *CXCL1*, *CXCL8* and *CCL20* mRNA expression was measured by RT-qPCR (A); *DNMT3B* mRNA levels in BEAS-2B cells incubated with heat killed PAK or medium control for 12 hours (B); Dnmt3b deficient (knock out, *DNMT3B* KO; open bars) and control (Ctr; black bars) BEAS-2B cells were stimulated with heat killed PAK (+) or medium control (-) for 12 hours. *CXCL1*, *CXCL8* and *CCL20* mRNA levels were measured by RT-qPCR (C) and corresponding protein levels in the supernatant by ELISA (D). Data are presented as means ± SEM (n = 4) and representative of two to three independent experiments and for two *DNMT3B* KO and control clones. *p < 0.05, **p < 0.01, *** p < 0.001.

To obtain a first insight into the role of Dnmt3b in the regulation of innate immune responses by respiratory epithelial cells, human bronchial BEAS-2B cells were pretreated with the pan DNA methyltransferase inhibitor RG108 [16] and stimulated with heat killed wild-type *P*. *aeruginosa* (PAK). RG108 at doses up to 50 μM did not affect cell viability (S1A Fig), but (at a dose of 10 μM) increased PAK-induced mRNA expression of the chemokines CXCL1, CXCL8 and CCL20 (Fig 1A). PAK stimulation decreased *DNMT3B* mRNA levels (Fig 1B) without affecting *DNMT3A* or *DNMT1* mRNA expression (S1B Fig). Since *P*. *aeruginosa* is a major pathogen in infections of lungs affected by cystic fibrosis, we then examined DNMT expression in the human cystic fibrosis bronchial epithelial cell line CFBE41o- complemented with the wild type cystic fibrosis transmembrane conductance regulator (*CFTR*) gene after *P*. *aeruginosa* infection for 1 hour using publicly available data (GSE30439) [17]. *DNMT3B* expression was significantly decreased by *P*. *aeruginosa* whilst the expression of *DNMT3A* or *DNMT1* was not affected (S1C Fig). To evaluate DNMT expression in 3 dimensional cultures of epithelial cells, we made use of human primary bronchial epithelial cells cultured in air liquid interface exposed to *P*. *aeruginosa* PAO1 for 24 hours in experiments published by our laboratory [18]. Consistent with the results obtained in cell lines, PA01 decreased *DNMT3B* but not *DNMT3A* or *DNMT1* expression (S1D Fig). In mouse primary airway epithelial cells grown in air liquid interface *Dnmt3b* as well as *Dnmt3a* and *Dnmt1* were decreased upon exposure to *P*. *aeruginosa* for 1 or 24 hours (GSE7957) [19] (S1E Fig). Together these data show that *DNMT3B* expression is decreased in human respiratory epithelial cell lines, as well as in primary human and mouse respiratory epithelial cells after exposure to *P*. *aeruginosa*. To determine the role of Dnmt3b in the responsiveness of respiratory epithelial cells to *P*. *aeruginosa*, we generated *DNMT3B* knockout BEAS-2B bronchial epithelial cells using CRISPR-Cas9; control BEAS-2B cells were generated in the same way using a non-targeting control guide RNA. Two confirmed Dnmt3b deficient and two control BEAS-2B clones (S1F Fig) were exposed to heat-killed PAK for 12 hours, after which mRNA and supernatants were harvested. PAK induced a marked upregulation of mRNA and protein levels of CXCL1, CXCL8 and CCL20, which were further increased in Dnmt3b deficient cells (Fig 1C and 1D). In contrast, the expression of genes encoding defensins (β-defensin 1 and 2), barrier function associated proteins (Tight junction protein 1 and 2) and cytokines (IL-1β and TNFα) was not altered by Dnmt3b deficiency (S1G Fig). Therefore, we focused on the regulation of chemokine production by Dnmt3b. Overexpression of Dnmt3b in BEAS-2B cells (S2A Fig) did not influence PAK-induced CXCL1, CXCL8 or CCL20 production (S2B Fig). Hence, these data suggest that endogenous Dnmt3b suppresses chemokine/cytokine production by bronchial epithelial cells upon activation by PAK *in vitro*.

### Dnmt3b inhibits *Pseudomonas aeruginosa* induced chemokine production by bronchial epithelial cells *in vitro* independent of flagellin expression

**Fig 2:**
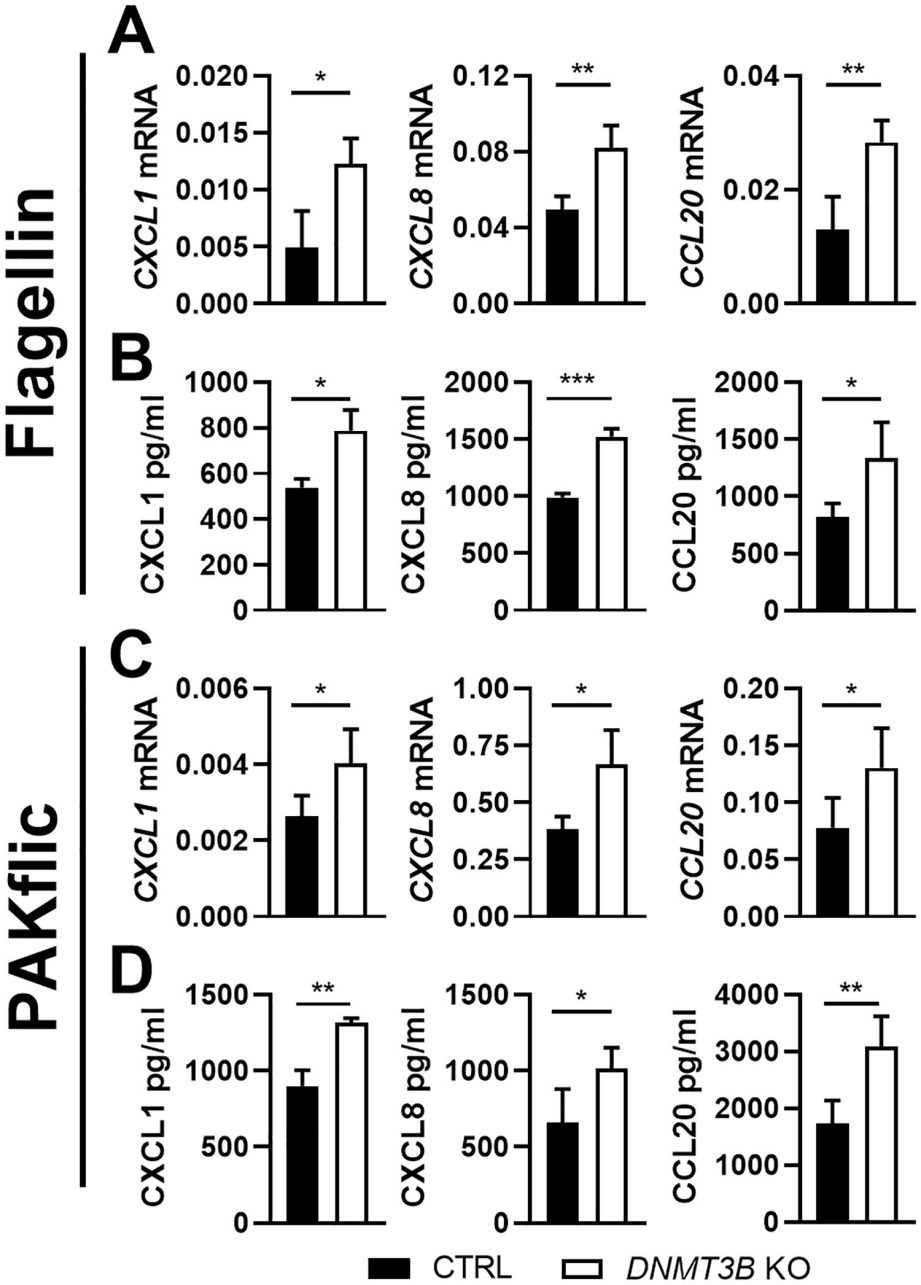
Dnmt3b deficiency in BEAS-2B bronchial epithelial cells promotes *Pseudomonas aeruginosa* induced chemokine production *in vitro* independent of flagellin. Dnmt3b deficient (knock out, *DNMT3B* KO; open bars) and control BEAS-2B cells (black bars) were stimulated with flagellin at final contrition of 1 μg/ml (panels A and B) or heat-killed flagellin-deficient PAK (PAKflic, MOI = 50, panels C and D) for 12 hours. *CXCL1*, *CXCL8* and *CCL20* mRNA levels were measured by RT-qPCR (A, C) and corresponding protein levels in supernatants by ELISA (B, D). Data are expressed as the mean ± SEM (n = 4) and representative of two to three independent experiments and for two *DNMT3B* KO and control clones. *p < 0.05, **p < 0.01, *** p < 0.001.

Flagellin is an important virulence factor expressed by PAK and a potent activator of respiratory epithelial cells [6]. Flagellin induced *CXCL1*, *CXCL8* and *CCL20* mRNA and protein expression by BEAS-2B cells, which was enhanced in Dnmt3b deficient cells (Fig 2A and 2B). To determine whether flagellin contributes to the regulatory function of Dnmt3b in respiratory epithelial cells activated by PAK, we stimulated BEAS-2B cells with flagellin deficient PAK (PAKflic). Similar to results obtained with wild-type PAK and purified flagellin, PAKflic induced more *CXCL1*, *CXCL8* and *CCL20* mRNA expression and protein production by Dnmt3b deficient BEAS-2B cells when compared with control BEAS-2B cells (Fig 2C and 2D). Overexpression of Dnmt3b in BEAS-2B cells did not influence CXCL1, CXCL8 or CCL20 production induced by flagellin or PAKflic (S2C Fig). The heating process used to kill PAK and PAKflic slightly reduced the biological activity of purified flagellin toward BEAS-2B cells, whereas trypsin totally abolished flagellin activity (S3 Fig), which is consistent with the reported heat stability of flagellin [20]. Together, these results suggest that although Dnmt3b influences flagellin responses, the role of Dnmt3b in PAK-induced chemokine production by BEAS-2B cells does not dependent on the presence of flagellin in this bacterium.

### Dnmt3b inhibits *Pseudomonas aeruginosa* induced NF-ĸB p65 binding to the *CXCL1* promoter in bronchial epithelial cells *in vitro*

**Fig 3:**
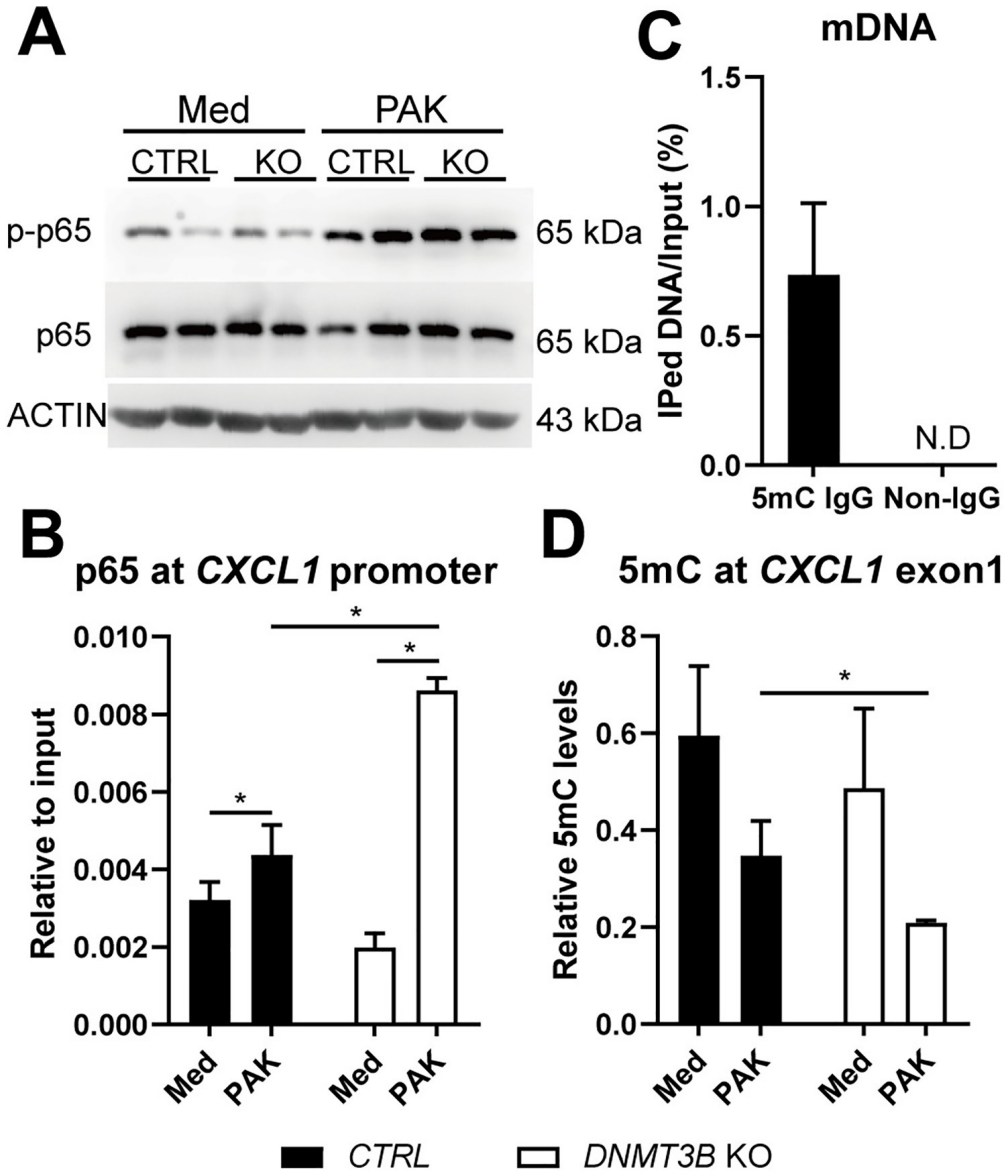
Dnmt3b inhibits *Pseudomonas aeruginosa* induced NF-ĸB p65 binding to the *CXCL1* promoter in BEAS-2B bronchial epithelial cells *in vitro*. Dnmt3b deficient (knock out, *DNMT3B* KO) and control BEAS-2B cells (CTRL) were stimulated with heat killed PAK at MOI of 50 for 30 min, total protein was extracted from the activated cells for detection of RelA/NF kappa B (NF-κB) p65 and Phospho-p65 (S536) by western blot (A). DNMT3B deficient (knock out, *DNMT3B* KO; open bars) and control BEAS-2B cells (CTRL; black bars) were stimulated with heat killed PAK (MOI = 50) for 1 hour, NF-κB p65 binding to *CXCL1* promoter was evaluated by ChIP (B). Control methylated DNA (mDNA) was used as control input for testing the efficiency of MeDIP and the specificity of 5-mC IgG, N.D = no signal detected (C). Dnmt3b deficient (knock out, *DNMT3B* KO) and control BEAS-2B cells (CTRL) were stimulated with heat killed PAK (MOI = 50) for 12 hours, MeDIP was performed to measure DNA methylation (5mC) levels at *CXCL1* exon1 (D). Data are expressed as the mean ± SEM (n = 4) and representative of two independent experiments. *p < 0.05.

The expression of genes modified by Dnmt3b deficiency in the experiments described above are highly regulated by NF-κB [21]. PAK induced NF-κB activation in BEAS-2B cells as indicated by increased phosphorylation of the NF-κB subunit p65, but this was not altered by Dnmt3b deficiency (Fig 3A). This led us to hypothesize that Dnmt3b might affect chemokine production downstream of NF-κB signaling. To this end, we performed chromatin immunoprecipitation (ChIP) to measure the binding of NF-κB to the promoter region of *CXCL1*, which is a necessary step to elicit NF-κB target gene expression [22]. PAK induced the binding of NF-κB to the promoter region of *CXCL1*, and deletion of *DNMT3B* further potentiated the effect (Fig 3B). It is well documented that DNA methylation regulates gene expression by changing chromatin accessibility to transcriptional factors [23], and Dnmt3b promotes *de novo* DNA methylation [12]. DNA methylation of the p65 binding motif at the promoter of *CXCL1* decreases *CXCL1* expression by reducing p65 binding [24]. To evaluate whether Dnmt3b influenced p65 binding via regulating DNA methylation of the binding sites at the regulatory elements of *CXCL1*, we performed methylated DNA immunoprecipitation (MeDIP) using a 5-methylcytosine antibody (Fig 3C). While control methylated DNA was successfully precipitated in this experiment, we could not detect a signal at the *CXCL1* promoter, suggesting low levels of DNA methylation in this region. Dnmt3b deficiency tended to reduce DNA methylation in exon 1 of *CXCL1* in unstimulated BEAS-2B cells; PAK induced a decrease in DNA methylation in this region, which was further reduced in Dnmt3b deficient cells (Fig 3D).

### Bronchial epithelial deficiency of Dnmt3b promotes CXCL1 production by bronchial epithelial cells in the early phase during *Pseudomonas* pneumonia *in vivo*

**Fig 4:**
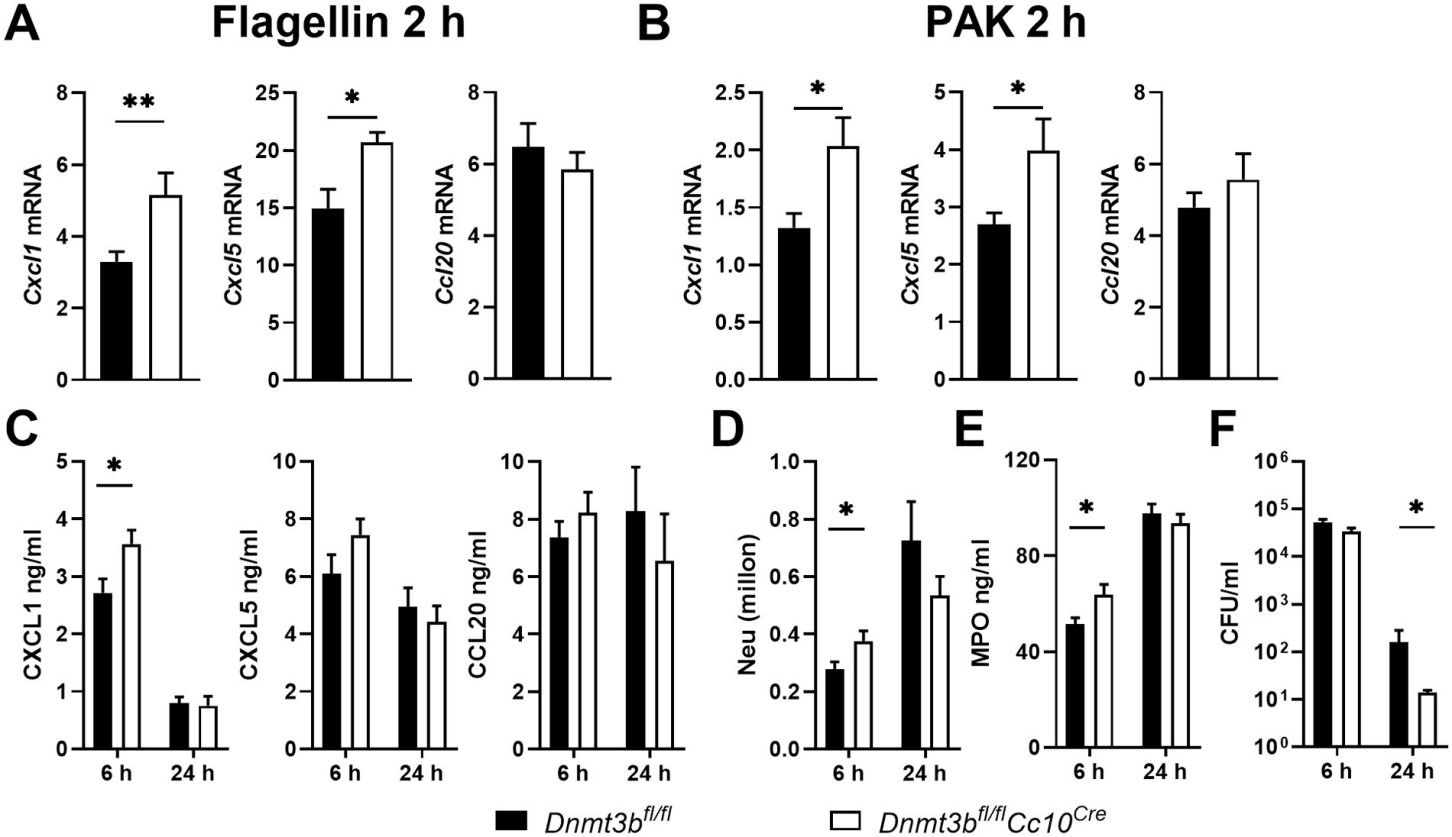
Bronchial epithelial deficiency of Dnmt3b promotes CXCL1 production by bronchial epithelial cells at the early phase of *Pseudomonas* pneumonia *in vivo*. *Dnmt3b*^*fl/fl*^*Cc10*^*Cre*^ and control *Dnmt3b*^*fl/fl*^ mice received flagellin (1 μg) purified from *P*. *aeruginosa* intranasally. *Cxcl1*, *Cxcl5* and *Ccl20* mRNA expression in bronchial brushes collected at 2 hours (A). *Dnmt3b*^*fl/fl*^*Cc10*^*Cre*^ and control *Dnmt3b*^*fl/fl*^ mice were infected with PAK (5 x 10^6^ CFU) intranasally. *Cxcl1*, *Cxcl5* and *Ccl20* mRNA expression in bronchial brushes collected at 2 hours (B). CXCL1, CXCL5 and CCL20 (C), neutrophil counts (D), MPO (E) and bacterial counts (F) in BALF were determined in BALF harvested after 6 or 24 hours. Data are presented as means ± SEM of 8 mice per group at each time point. * p < 0.05.

To investigate the role of Dnmt3b in bronchiolar epithelial cells during *Pseudomonas* pneumonia *in vivo*, we crossed mice in which the *Dnmt3b* gene is flanked by two lox-P sites (*Dnmt3b*^*fl/fl*^ mice) with mice expressing Cre-recombinase under the control of the club cell 10 kD (CC10) promoter (*Cc10*^*Cre*^ mice) to generate *Dnmt3b*^*fl/fl*^*Cc10*^*Cre*^ mice. Our laboratory previously showed that Cre-recombinase is specifically active in bronchiolar epithelial cells in *Cc10*^*Cre*^ mice [25]; thus, the cellular distribution of Dnmt3b deficiency in *Dnmt3b*^*fl/fl*^*Cc10*^*Cre*^ mice corresponds with the *in vitro* studies using Dnmt3b deficient bronchial epithelial cells described above. We first sought to establish whether Dnmt3b influences flagellin induced chemokine expression in bronchial epithelial cells *in vivo*. Bronchial brushes highly expressed mRNAs encoding the epithelial cell marker CD326 and the bronchial epithelial cell marker CC10, while leukocytes collected by bronchoalveolar lavage (BAL) expressed high levels of mRNA encoding CD45 (S4 Fig), indicating a high purity of respiratory epithelial cells in the brushes. *Dnmt3b*^*fl/fl*^*Cc10*^*Cre*^ mice, relative to *Dnmt3b*^*fl/fl*^ Cre-negative littermate control mice, showed significantly enhanced *Cxcl1* and *Cxcl5* expression in bronchial brushes harvested 2 hours after flagellin treatment, while *Ccl20* expression was unaffected (Fig 4A). To determine the impact of bronchial epithelial Dnmt3b on host defense during *Pseudomonas* pneumonia, *Dnmt3b*^*fl/fl*^*Cc10*^*Cre*^ and *Dnmt3b*^*fl/fl*^ control mice were infected with (wild-type) PAK via the airways and euthanized 2, 6 or 24 hours thereafter for analysis. In agreement with the results obtained after flagellin administration, bronchial brushes collected from *Dnmt3b*^*fl/fl*^*Cc10*^*Cre*^ mice 2 hours after PAK infection expressed higher *Cxcl1* and *Cxcl5* mRNA levels compared to those from *Dnmt3b*^*fl/fl*^ control mice, while *Ccl20* mRNA were similar between groups (Fig 4B). PAK elicited high levels of CXCL1, CXCL5, and CCL20 in BAL fluid (BALF) at 6 hours post-infection, with lower concentrations at 24 hours (Fig 4C). *Dnmt3b*^*fl/fl*^*Cc10*^*Cre*^ mice showed higher levels of CXCL1 when compared with littermate control mice at 6 hours after infection; this difference was not present anymore at 24 hours. CXCL5 levels tended to be higher in BALF of *Dnmt3b*^*fl/fl*^*Cc10*^*Cre*^ mice relative to control mice at 6 hours post-infection (P = 0.07); BALF CCL20 concentrations were similar in *Dnmt3b*^*fl/fl*^*Cc10*^*Cre*^ and control mice (Fig 4C).

### Bronchial epithelial deficiency of Dnmt3b promotes early neutrophil recruitment and bacterial clearance during *Pseudomonas* pneumonia *in vivo*

Neutrophils are attracted to the airways by locally released CXC chemokines such as CXCL1 and play a key role in host defense during pneumonia [26]. In agreement with higher local CXCL1 concentrations, *Dnmt3b*^*fl/fl*^*Cc10*^*Cre*^ mice had higher neutrophil numbers in their BALF when compared with control mice at 6 hours after infection with PAK (Fig 4D). Additionally, *Dnmt3b*^*fl/fl*^*Cc10*^*Cre*^ mice had higher concentrations of the neutrophil degranulation product myeloperoxidase in BALF when compared with control mice (Fig 4E). Neutrophils are important for clearance of *Pseudomonas* from the airways [27,28]. In agreement with increased neutrophil recruitment to the site of infection, *Dnmt3b*^*fl/fl*^*Cc10*^*Cre*^ mice demonstrated an accelerated bacterial clearance as reflected by lower bacterial burdens in BALF at 24 hours after infection when compared with control mice (Fig 4F). The extent of PAK induced lung pathology, as determined by semi-quantitative scoring of hematoxylin and eosin (H&E) stained lung slides, did not differ between *Dnmt3b*^*fl/fl*^*Cc10*^*Cre*^ and control mice (S5A and S5B Fig). Likewise, bronchial Dnmt3b deficiency did not impact IL-1β and TNFα release in BALF during *Pseudomonas* pneumonia (S5C Fig).

### The enhanced pulmonary response of mice with bronchial epithelial Dnmt3b deficiency to *Pseudomonas* depends on bacterial expression of flagellin

**Fig 5:**
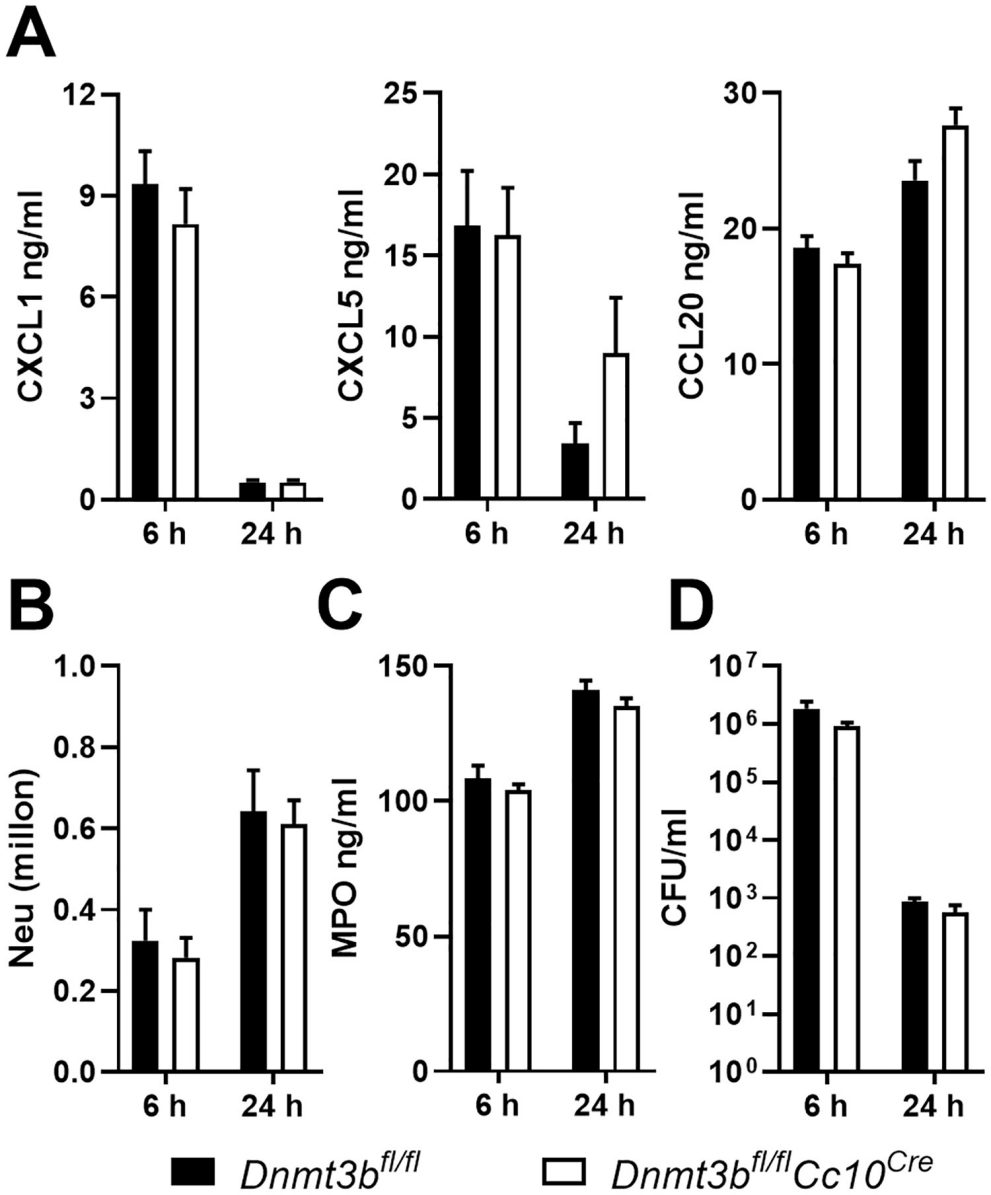
Bronchial epithelial deficiency of Dnmt3b does not modify pulmonary responses during pneumonia caused by flagellin-deficient *Pseudomonas*. *Dnmt3b*^*fl/fl*^*Cc10*^*Cre*^ and control *Dnmt3b*^*fl/fl*^ mice were infected with flagellin-deficient PAK (PAKflic, 5 x 10^6^ CFU) intranasally. CXCL1, CXCL5 and CCL20 were determined in BALF harvested after 6 or 24 hours (A). Neutrophil counts (B), MPO (C) and bacterial counts (D) in BALF were determined. Data are presented as means ± SEM of 8 mice per group at each time point.

We and others have previously shown that activation of respiratory epithelial cells by flagellin drives protective innate immunity during *Pseudomonas* pneumonia through the induction of TLR5-MyD88 dependent signaling [7,8,9]. To determine a role for flagellin in Dnmt3b-mediated inhibition of the immune response in bronchiolar epithelial cells and bacterial clearance during *Pseudomonas* infection *in vivo*, we infected *Dnmt3b*^*fl/fl*^*Cc10*^*Cre*^ and control mice with PAKflic via the airways. Unlike after infection with wild-type PAK *in vivo* (Fig 4) or stimulation with PAKfilc *in vitro* (Fig 2), *Dnmt3b*^*fl/fl*^*Cc10*^*Cre*^ mice infected with PAKflic showed no differences with regard to local chemokine release, neutrophil recruitment or bacterial clearance (Fig 5). Bronchial epithelial Dnmt3b deficiency also did not influence local IL-1β or TNFα release, or the extent of lung pathology (S6 Fig). Taken together, these results suggest that bronchial epithelial Dnmt3b dampens mucosal immunity during *Pseudomonas* airway infection resulting in a reduced bacterial clearance in mice, which is dependent on Dnmt3b mediated modulation of respiratory epithelial responses induced by the *Pseudomonas* component flagellin.

### Bronchial epithelial Dnmt3b deficiency does not affect the host response during pneumonia caused by *Klebsiella pneumoniae*

To further examine the role of flagellin in Dnmt3b regulated functions during bacterial pneumonia we infected *Dnmt3b*^*fl/fl*^*Cc10*^*Cre*^ and control mice with *Klebsiella pneumoniae*, an un-flagellated gram-negative bacterium. CXCL1 levels, neutrophil counts, MPO concentrations and bacterial burdens in BALF were not affected by Dnmt3b deficiency in bronchial epithelial cells after infection with *Klebsiella* (S7A, S7B, S7C and S7D Fig). Moreover, bacterial loads in extrapulmonary organs such liver and spleen, and in blood were comparable between groups (S7E Fig). These data provide further support for a role for flagellin in Dnmt3b mediated mucosal immune responses in the infected airways.

### Type II alveolar epithelial cell Dnmt3b does not influence the pulmonary response during *Pseudomonas* pneumonia *in vivo*

**Fig 6:**
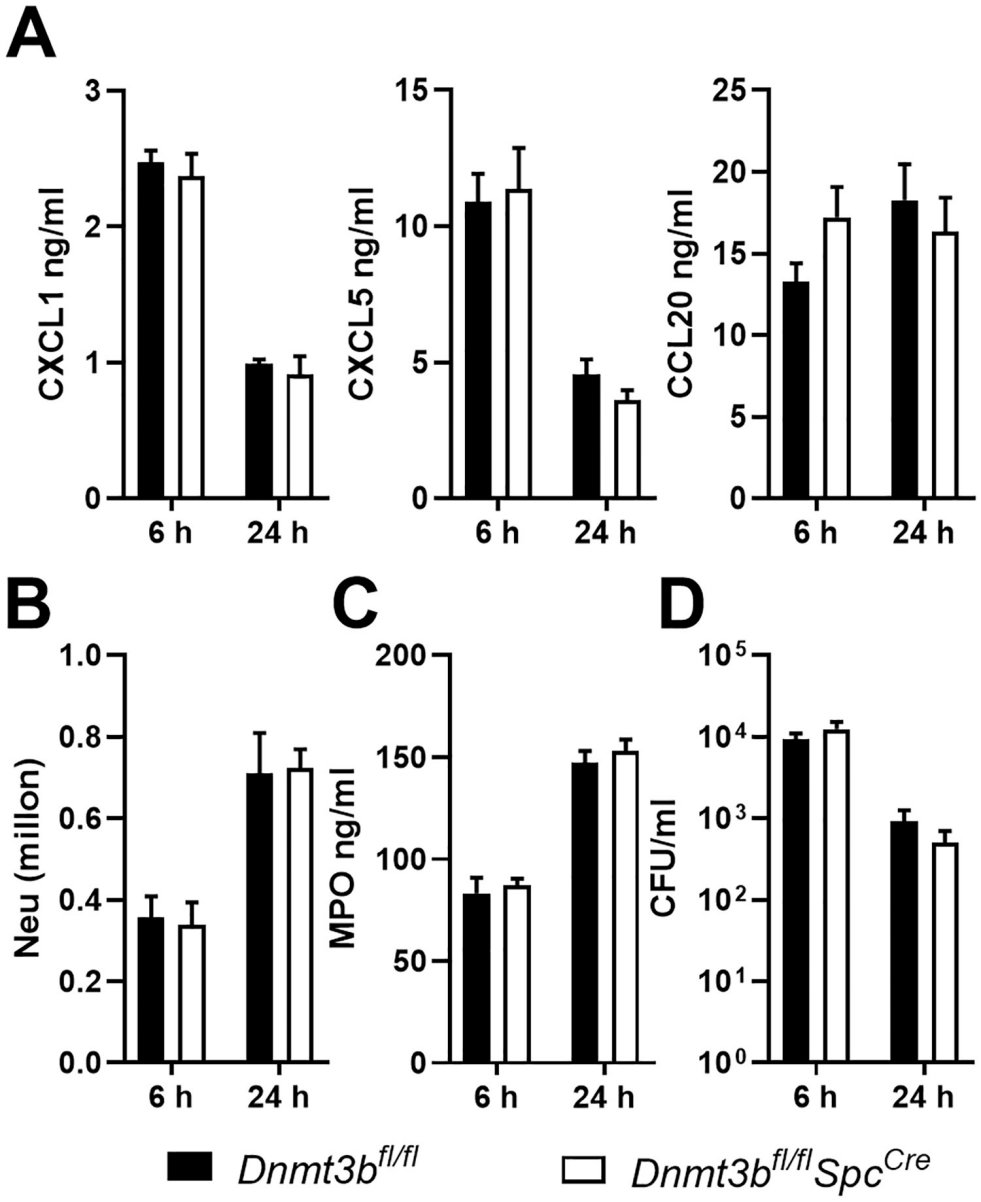
Type II alveolar epithelial cell Dnmt3b has no role in the pulmonary response during *Pseudomonas* pneumonia *in vivo*. *Dnmt3b*^*fl/fl*^*SpC*^*Cre*^ and control *Dnmt3b*^*fl/fl*^ mice were infected with PAK (5 x 10^6^ CFU) intranasally. CXCL1, CXCL5 and CCL20 were determined in BALF harvested after 6 or 24 hours (A). Neutrophil counts (B), MPO (C) and bacterial counts (D) in BALF were determined. Data are presented as means ± SEM of 8 mice per group at each time point.

Type II alveolar epithelial cells (AEC2) have been implicated in host defense during *P*. *aeruginosa* infection [29]. To determine whether the role of Dnmt3b in the host response during *Pseudomonas* pneumonia is restricted to bronchiolar epithelial cells, we crossed *Dnmt3b*^*fl/fl*^ mice with mice expressing Cre-recombinase under the control of the surfactant protein C promoter (*SpC*^*cre*^ mice) to generate AEC2 specific Dntm3b deficient (*Dnmt3b*^*fl/fl*^ *SpC*^*cre*^) mice. We previously showed that in *SpC*^*cre*^ mice Cre-recombinase is specifically active in AEC2 [25]. In contrast to *Dnmt3b*^*fl/fl*^*Cc10*^*Cre*^ mice, *Dnmt3b*^*fl/fl*^*SpC*^*cre*^ mice did not differ from littermate control mice with regard to local release of CXCL1 (Fig 6A), neutrophil recruitment (Fig 6B), BALF MPO levels (Fig 6C) or bacterial clearance (Fig 6D) after infection with wild-type PAK via the airways. Furthermore, *Dnmt3b*^*fl/fl*^*SpC*^*cre*^ and control mice showed similar CXCL5, CCL20 (Fig 6A), IL-1β and TNF-α BALF levels upon infection with PAK (S8 Fig). These data suggest that Dnmt3b expressed in AEC2 does not influence innate immune mechanisms during *Pseudomonas* pneumonia.

## Discussion

Here we report that deficiency of *DNMT3B*, the gene encoding a key enzyme involved in *de novo* DNA methylation, in human bronchial epithelial cells *in vitro* results in increased production of chemokines implicated in mucosal immunity, including CXCL1, in response to the common human pathogen *P*. *aeruginosa*. This role of Dnmt3b in epithelial CXCL1 production was reproduced *in vivo*, using mice with bronchial epithelial cell specific deletion of Dnmt3b infected with *P*. *aeruginosa* via the airways. In these mice, increased CXCL1 release into the airways was associated with enhanced neutrophil recruitment and an accelerated bacterial clearance, which depended on expression of flagellin by *Pseudomonas*. Mechanistically, *DNMT3B* deficiency was shown to decrease DNA methylation levels at NF-κB binding regions in the promoter region of *CXCL1*, allowing increased NF-κB binding and transcription of NF-kB response genes upon exposure to *Pseudomonas*.

Epithelial cells are the first line of defense in lung innate immunity during pulmonary infection [4]. One of their key functions is to produce chemokines that recruit innate immune cells such as neutrophils to control the infection. Previous studies documented that the Dnmt inhibitor 5′-azacytidine increased *CCL20* expression in human gingival epithelial cells in response to *Fusobacterium nucleatum* [30], as well as *Cxcl1* expression in mouse MLE-12 respiratory epithelial cells stimulated with IL-17 [31]. Our current finding that the Dnmt inhibitor RG108 enhanced *CXCL1*, *CXCL8* and *CCL20* expression in BEAS-2B cells stimulated with *P*. *aeruginosa* further supports a role for Dnmt’s in the induction of chemokines by respiratory epithelial cells. The effect of RG108 could be reproduced by elimination of *DNMT3B* in BEAS-2B cells, and Dnmt3b deficient BEAS-2B cells also produced more CXCL1, CXCL8 and CCL20 upon stimulation with the *Pseudomonas* component flagellin. The expression of chemokines is partly regulated by NF-κB and binding of the NF-κB subunit p65 to chemokine promoter regions drives their gene transcription [32]. The ability of NF-κB to bind DNA and to trigger inflammatory gene transcription is determined at least in part by the extent of DNA methylation [33]. *DNMT3B* was downregulated after activation of BEAS-2B cells by PAK, which coincided with a decrease in methylation at exon 1 of *CXCL1*. Deletion of *DNMT3B* in BEAS-2B cells further decreased methylation at exon 1 of *CXCL1* while enhancing p65 binding to the *CXCL1* promoter, providing insight into the mechanism by which Dnmt3b regulates the expression of *CXCL1*. Noteworthy, DNA methylation can affect other types of epigenetic regulation, such as that mediated by Polycomb-Repressive Complexes (PRCs) [34]. Although PRCs are particularly associated with development, differentiation, and stem cell renewal [35], some studies suggested that PRCs regulate the expression of chemokines, such as CXCL9 and CXCL10 [36]. Therefore, we cannot rule out the possible involvement of PRCs in the regulation of CXCL1 expression in Dnmt3b deficient cells. Additionally, Dnmt3a and Dnmt1 are involved in de novo DNA methylation and maintaining methylation, respectively [23]. Dnmt3b has been reported to work synergistically with Dnmt3a, and their combined effects on *de novo* methylation have been demonstrated during the epigenetic regulation of hematopoietic stem cell fate decisions [37]. Therefore, further investigations are needed to determine whether complementary mechanisms mediated by Dnmt3a occur in Dnmt3b deficient cells during bacterial infection. The existence of such complementary mechanisms could explain the fact that Dnmt3 deficiency only affected the early response to *Pseudomonas* infection of the airways. Other factors might also be involved in the transient effect of Dntm3b deficiency, including later induction of other host response mediators by *Pseudomonas* that overrule the effect of Dnmt3b deficiency and the use of a “single-injury” model with clearance of bacteria (i.e., possibly, repeated injuries might reveal a more sustained role for Dnmt3b).

The role of bronchial epithelial Dnmt3b in limiting *Pseudomonas* induced *CXCL1* transcription in human epithelial cells *in vitro* was confirmed in *Dnmt3b*^*fl/fl*^*Cc10*^*Cre*^ mice *in vivo*, in which enhanced CXCL1 mRNA levels in bronchial brushes (2 hours) associated with elevated CXCL1 levels in BALF early (6 hours) after infection. In *Dnmt3b*^*fl/fl*^*Cc10*^*Cre*^ mice Cre-mediated deletion of *Dnmt3b* is restricted to CC10+ cells, which are the major cell type located in mouse upper airways, trachea and bronchial regions [38]. We showed that bronchial brushes harvested from mice are highly enriched for CC10+ cells, confirming previous reports [39,40]. CC10+ cells are an important source of chemokines [40], which can explain why increased *Cxcl1* transcription in bronchial brush cells can result in increased CXCL1 protein levels in BALF. Notably, although bronchial CXCL5 mRNA levels were higher in *Dnmt3b*^*fl/fl*^*Cc10*^*Cre*^ than in control mice infected with PAK, CXCL5 protein levels in BALF were not significantly increased. Moreover, at 24 hours after infection, CXCL1 and CXCL5 concentrations in BALF were similar in both mouse strains. In addition to epithelial cells, these chemokines can be produced by myeloid cells, which likely can obscure possible differences in chemokine release by epithelial cells at later stages during the infection, i.e., after influx of neutrophils. Nonetheless, the enhanced CXCL1 release early after infection was accompanied by a faster influx of neutrophils into the alveolar compartment, which is a likely explanation for a subsequent accelerated bacterial clearance [27,28]. Indeed, neutrophils are among the first cells recruited to infectious sites and are crucial for controlling *P*. *aeruginosa* infection through various mechanisms including degranulation, phagocytosis, and the generation of neutrophil extracellular traps [41].

We used BEAS-2B cells to study the function of Dnmt3b in human respiratory epithelial cells. While this cell line is frequently used for mechanistic studies considering its convenience for genetic modification, it is worth noting that BEAS-2B cells have several limitations including their inability to form barriers [42] and to produce mucus [43]. Primary respiratory epithelial cells are not easily accessible for genetic manipulation. We here do provide evidence that the expression of *DNMT3B* (*Dnmt3b*) is regulated in the same way (reduced) in human and mouse primary epithelial cells, grown in air-liquid interface, as in BEAS-2B cells upon exposure to *Pseudomonas*. The data obtained with Dnmt3b deficient BEAS-2B cells and *Dnmt3b*^*fl/fl*^*Cc10*^*Cre*^ mice differed in some aspects. First, expression of *CCL20* induced by PAK was affected by Dnmt3b deficiency in BEAS-2B cells but not in bronchial brushes from *Dnmt3b*^*fl/fl*^*Cc10*^*Cre*^ mice, which could be due to differences relating to species (human versus mouse), cell type (cell line versus primary cells) and/or stimulation (heat-killed PAK stably present in cell culture medium versus a gradually decreasing burden of viable bacteria). Importantly, whereas flagellin was not required for the role of Dnmt3b in *Pseudomonas* induced *CXCL1* expression in BEAS-2B cells *in vitro*, *Dnmt3b*^*fl/fl*^*Cc10*^*Cre*^ mice only demonstrated an enhanced innate immune response after infection with WT PAK but not after infection with flagellin deficient PAKflic. While flagellin activates TLR5, intact *P*. *aeruginosa* can also activate TLR2, 4 and 9 [44]; these TLRs are expressed by BEAS-2B cells [45] and activation of different TLRs induces a common set of genes including *CXCL1* downstream of NF-κB signaling [46,47]. In BEAS-2B cell cultures, cells were continuously exposed to PAK for 10 hours, likely allowing stimulation of multiple TLRs, which may compensate for the lack of flagellin-TLR5 signaling upon exposure to PAKflic. In mice, however, the recognition of flagellin by TLR5 is required for airway epithelial cells to sense *P*. *aeruginosa* and to clear the infection [9,48,49,50,51], which may explain the prominent role of flagellin in the effect of bronchial epithelial Dnmt3b in regulating the mucosal immune response during *Pseudomonas* infection *in vivo*. In line with this, we found that the number of PAKflic CFU was higher than that of PAK at 6 hours after infection, which might explain higher IL-1β and TNFα in BALF of PAKflic infected mice as compared to mice infected with PAK. Bronchial epithelial Dnmt3b played no role in CXCL1 release, neutrophil recruitment or anti-bacterial defense during *K*. *pneumoniae* infection, which–considering that this gram-negative pathogen does not express flagellin–further supports a role for flagellin in Dnmt3b mediated functions in the respiratory epithelium. An additional explanation for dissimilarities between BEAS-2B cells *in vitro* and mice *in vivo* could be species differences; in this context studies comparing the responsiveness of mouse respiratory epithelial cells to PAK and PAKflic would be of interest.

Dnmt3b ablation in mouse AEC2 did not impact CXCL1 production, neutrophil influx or bacterial clearance after infection with *Pseudomonas*. The differential roles of Dnmt3b in bronchial epithelial (Cc10) cells and AEC2 (SPC cells) could have several mutually non-exclusive explanations. First, AEC2 likely are less important for the production of chemoattractant mediators like CXCL1, but rather are crucial for the synthesis of surfactant lipids and proteins required for the reduction of surface tension in order to prevent collapse of the lungs [4]. Thus, AEC2 produced CXCL1 likely accounts for only a minor part of CXCL1 released in BALF and a possible effect of Dnmt3b on CXCL1 production by AEC2 might not impact overall CXCL1 production. Second, Dnmt3b might function differently in these two epithelial cell types. Notably, whilst the cell-specific expression of the *Cc10* (bronchial) and *Spc* (AEC2) promoters has been documented by several laboratories including ours [25], a small population of epithelial cells named bronchioalveolar stem cells, located at the bronchioalveolar-duct junctions, express both *Cc10* and *Spc* [52,53]. Our data do not exclude a role for Dnmt3b in this cell population during *Pseudomonas* pneumonia.

Our data do not provide insight into the potential importance of Dnmt3b as a therapeutic target during *Pseudomonas* pneumonia. Translation of the current respiratory epithelial cell-specific results on Dnmt3b function to a possible therapeutic targeting this enzyme requires additional investigations. This also holds true for further studies on the use of flagellin, administered via the airways as a possible immune enhancing strategy in the treatment of respiratory tract infections, and the role of Dnmt3b herein [6].

Changes in DNA methylation in respiratory epithelia have been implicated in several inflammatory lung diseases, including asthma, chronic obstructive pulmonary disease, cystic fibrosis and idiopathic pulmonary fibrosis [54,55,56]. Knowledge of a potential role of epithelial DNA methylation modifications in the host response to respiratory pathogens is highly limited. This study provides evidence for a role of Dnmt3b, an enzyme mediating *de novo* DNA methylation, in bronchial epithelial cells in regulating the early innate immune response during airway infection caused by *P*. *aeruginosa*.

## Methods

### Ethics statement

All mouse experiments were approved by the Institutional Animal Care and Use Committee of the University of Amsterdam.

### Mice

Homozygous *Dnmt3b*^*fl/lf*^ mice (RBRC03733, RIKEN BRC, Tsukuba, Japan) [57] were crossed with mice expressing cre recombinase under the control of the club cell 10-kD promoter (*Cc10*^*Cre*^ mice) [25] to generate bronchiolar epithelial–specific Dnmt3b-deficient (*Dnmt3b*^*fl/fl*^*Cc10*^*Cre*^) mice or with mice expressing cre recombinase under the control of the surfactant protein C promoter (*SpC*^*Cre*^ mice) [9] to generate type II alveolar epithelial–specific Dnmt3b-deficient (*Dnmt3b*^*fl/fl*^*SpC*^*Cre*^) mice. *Dnmt3b*^*fl/fl*^ Cre–negative littermates were used as controls in all experiments. All genetically modified mice were backcrossed at least eight times to a C57Bl/6 background and age and sex matched when used in experiments. Mice were used at 8–12 weeks of age.

### Bacterial strains and culture conditions

Wild-type *P*. *aeruginosa* PAK, flagellin deficient *P*. *aeruginosa* PAKflic [58] and *Klebsiella pneumoniae* serotype 2 (American Type Culture Collection no. 43816) were cultured as described previously [9,59]. Briefly, PAK and PAKflic were grown to mid-logarithmic phase in Luria broth at 37°C with shaking and *Klebsiella pneumoniae* was cultured to mid-logarithmic phase in Tryptic Soy Broth. Bacteria were then harvested by centrifugation at 3,000 rpm for 10 minutes. After washing twice with pyrogen-free 0.9% NaCl, bacteria were suspended in 10 ml of 0.9% NaCl, the number of bacteria was determined by serial dilution in sterile isotonic saline and culture on blood agar plates. Bacteria were diluted to 10^8^ colony-forming units (CFU)/ml for later use.

### Cells

The human bronchial epithelial cell line BEAS-2B [60] was obtained from the American Type Culture Collection (ATCC, Rockville, MD). Cells were cultured in DMEM (InvivoGen, San Diego, CA) supplemented with 10% FBS and 100 U/ml penicillin and 100 μg/ml streptomycin (Invitrogen, San Diego, CA) and kept at 37°C in an incubator with a humidified atmosphere containing 5% CO_2_. BEAS-2B cells were stimulated with heat-killed (65 °C for 15 minutes) wild-type *P*. *aeruginosa* (PAK), isogenic flagellin deficient *P*. *aeruginosa* (PAKflic) (both at multiplicity of infection (MOI) = 50) or flagellin purified from *P*. *aeruginosa* (tlrl-pafla, Invivogen, San Diego, CA; 1 μg/ml) for 12 hours. Before stimulation cells were plated in 24-well plates overnight. A lentivirus mediated CRISPR/Cas9 system was used to generate *DNMT3B* knockout cells as described [61]. Briefly, the single guide (sg)RNA targeting sequences were designed using an online gRNA design tool (Crispr.mit.edu). The sgRNA sequence 5-ATCCGCACCCCGGAGATCAG-3 was chosen to target *DNMT3B* and cloned into lentiCRISPR v2 (Addgene #52961, http://n2t.net/addgene:52961; RRID:Addgene_52961) [62]. Control cells were generated using the same method with a non-targeting sgRNA sequence ACGGAGGCTAAGCGTCGCAA. Two independent Dnmt3b deficient or control cell lines were selected for further experiments. Overexpression of Dnmt3b in BEAS-2B cells was performed using human *DNMT3B* ORF clone lentiviral particle (Vigene Biosciences, Rockville, MD) according to manufacturer’s instruction. Clones with confirmed Dnmt3b overexpression (by Western blot: see below) were selected for experiments; control clones were generated by transducing a lentiviral particle carrying the same vector skeleton used for Dnmt3b overexpression. For some experiments, cells were pretreated with the Dnmt inhibitor RG108 at 10 μM/ml [16] (Sigma, Zwijndrecht, Nederland) or vehicle DMSO for 12 hours, and then stimulated with heat killed bacteria at MOI = 50. The effect of RG108 treatment on cell viability was determined using flow cytometry after straining of cells with fixable viability dye eFluor 780 (Invitrogen, Carlsbad, CA).

### Western blot

Total protein was extracted from BEAS-2B cells, separated by 10% SDS gel electrophoresis and transferred to a PVDF membrane (Millipore, Billerica, MA). Membranes were blocked for 1 hour in 5% milk in tris-buffered saline with 0.1% Tween 20 (TBST) buffer (TBST) and incubated overnight with (primary) antibodies against phospho-NF-κB p65 (Ser536) (1: 1000, #3033; Cell Signaling Technology, Leiden, The Netherlands), NF-κB p65 (1: 1000, #3034N; Cell Signaling Technology, Leiden, The Netherlands), Dnmt3b (1:250, ab2851; Abcam, Cambridge, UK) or beta-actin (1: 1000, 4967L; Cell Signaling Technology) at 4°C. After incubation with horseradish peroxidase (HRP)-conjugated secondary antibody against rabbit IgG (1: 2000, #7074; Cell Signaling Technology) for 1 hour at room temperature, blots were imaged using Lumilight plus ECL substrate (Roche, Almere, The Netherlands) on an ImageQuant LAS 4000 biomolecular imager (GE Healthcare, Buckinghamshire, UK). For quantification, densitometry was performed with ImageJ (National Institutes of Health, Bethesda, MD; https://imagej.nih.gov/ij/) using the histogram function in a selected area of mean gray value for each band.

### Quantitative reverse transcription PCR (qRT-PCR)

Total RNA from BEAS-2B cells, mouse bronchial brushes and BALF cells was isolated with NucleoSpin columns (Bioke, Leiden, The Netherlands) according the manufacturer’s recommendations. All RNA samples were quantified by spectrophotometry and stored at -80°C until further analysis. cDNA was prepared using AMV Reverse Transcriptase (Promega, Leiden, The Netherlands) according to manufacturer’s instructions. Gene expression analysis was performed using a Roche LightCycler 480 thermocycler with SensiFAST Real-time PCR kit (#CSA-01190; Bioline, London, UK) using the gene specific primers listed in S1 Table. For qPCR of bronchial brush samples, primers specific for *Epcam* (encoding the epithelial cell marker CD326), *Ptprc* (hematopoietic cell marker CD45) and *Pecam1* (endothelial cell marker CD31) were used to quantify the enrichment of epithelial cells in the brushes; primers for *Scgb1a1* (cube cell marker CC10) were used to evaluate the relative enrichment of bronchial epithelial cells in the brushes. Data was analyzed with LinRegPCR based on PCR efficiency values derived from amplification curves [63]. All results were normalized to *Hprt* expression levels.

### Enzyme-linked immunosorbent assay (ELISA)

Human chemokine (C-X-C motif) ligand (CXCL)1, CXCL8, and chemokine (C-C motif) ligand (CCL) 20, as well as murine CXCL1, CXCL5, CCL20, tumor necrosis factor (TNF)-α, IL-1β and myeloperoxidase (MPO) were measured by species specific commercially available ELISA’s (R&D Systems, Minneapolis, MN) according to manufacturer’s description (protocols can be found at www.rndsystems.com).

### Methylated DNA immunoprecipitation (MeDIP)

MeDIP analysis was performed using Methylamp Methylated DNA Capture Kit (Epigentek, Farmingdale, NY) following the manufacturer’s instructions. BEAS-2B cells were stimulated with heat killed PAK for 12 hours. Cells were collected for DNA purification using DNeasy Blood & Tissue Kit (Qiagen, Hilden, Germany). Prior to immunoprecipitation, genomic DNA was sonicated with a Diagnode BioRuptor to obtain DNA fragments ranging in size from 200 to 1000 bp, with a mean fragment size of around 300 bp. Methylated DNA was captured using Methylamp Methylated DNA Capture Kit. In total 100 ng of fragmented DNA was applied in every antibody-coated well and incubated at room temperature on a horizontal shaker for 2 hours. The immunoprecipitated DNA was released by proteinase K. The DNA was eluted and adjusted to a final volume of 100 μl with nuclease-free water. For each sample, an input vial was included using total sonicated DNA as loading control. Total DNA and immunoprecipitated DNA were used to perform qPCR with *CXCL1* promoter primer pairs that included a p65 binding site (S2 Table).

### Chromatin immunoprecipitation (ChIP)

ChIP analysis was performed using ChIP-IT Express kit (Active Motif, Carlsbad, CA) following the manufacturer’s instructions. BEAS-2B cells were stimulated with heat killed PAK for 60 minutes and subsequently were cross-linked with 1% paraformaldehyde for 10 minutes at room temperature. Extracted chromatin was sheared by sonication using a Diagnode BioRuptor (10 pulses of 20 seconds each, with a 30 second rest between each pulse) into fragments of 200–800 bp length and immunoprecipitated using 3 μg NF-κB p65 antibody (Sigma-Aldrich, St. Louis, MO) at 4°C overnight. The samples were reverse cross-linked, and then proteins were digested with proteinase K and participated DNA was purified by NucleoSpin Gel and PCR Clean-up kit (Bioke, Leiden, The Netherlands) after reversal of cross-linking. qPCR amplification using 3 μl of total DNA and immunoprecipitated DNA was performed with the same primer pairs used in the MeDIP experiment (S2 Table).

### Induction of pneumonia and sampling of organs

To induce pneumonia, mice were inoculated with viable PAK, PAKflic (5 × 10^6^ CFU) or *K*. *pneumoniae* (1 × 10^4^ CFU) or flagellin purified from *P*. *aeruginosa* (1 μg, tlrl-pafla, Invivogen) intranasally. At predefined time points mice were euthanized by heart puncture after injection of ketamine/medotomidine as described [59,64]. Briefly, the right lung was used for bronchoalveolar lavage (BAL) by instilling 2 × 0.5 ml of sterile phosphate-buffered saline; the left lung was preserved for histopathology after fixation in 10% formalin. Bronchial brushing was performed after BAL to collect bronchial epithelial cells as described [40]; BALF was serially diluted and plated on blood agar plates for measurements of bacterial numbers. Cell counts in BALF were determined using a hemocytometer (Beckman Coulter, Fullerton, CA). BALF supernatants and bronchial brushes were stored at −20°C until further analysis. In some experiments BALF was pelleted and cells were lysed for RNA isolation. For all animal experiments, littermate controls and conditional knockout were mixed in cages (i.e. randomized) and procedures were done without knowledge of the genotype (blinded). Readout parameters were thereby determined in a double-blinded manner.

### Flow cytometry

Neutrophils in BALF were determined by flow cytometry as described [65]. Briefly, BALF cells were resuspended FACS buffer (5% BSA, 0.35 mM EDTA, 0.01% NaN3) and stained with fixable viability dye eFluor 780, rat anti mouse-CD45 PE-eFluor610 (30-F11), rat anti-mouse CD11b PE-Cy7 (clone M1/70), rat anti-mouse Siglec-F Alexa Fluor 647 (clone E50-2440), rat anti-mouse Ly-6C Alexa Fluor 700 (clone AL-21) (all from BD Biosciences) and rat anti-mouse Ly-6G FITC (clone 1A8; Biolegend, San Diego, CA) before loading on FACS Calibur (Becton Dickinson, Franklin Lakes, NJ). Data were analyzed using FlowJo software (Becton Dickinson). Neutrophils were identified as CD45+/Siglec-F-/CD11b+/Ly6C+/Ly6G+ cells. Examples of the gating strategy for BALF neutrophils are depicted in S9 Fig.

### Pathology scores

The left lung lobe was fixed in 10% formaldehyde solution and embedded into paraffin blocks. Sections were stained with Hematoxylin and eosin (H&E). Slides were coded and scored from 0 (absent) to 4 (severe) for the following parameters: interstitial inflammation, endothelialitis, bronchitis, edema, thrombi, pleuritis, and percentage of the lung surface demonstrating confluent (diffuse) inflammatory infiltrate by a pathologist blinded for groups. The total “lung inflammation score” was expressed as the sum of the scores for each parameter [66].

### Statistical analysis

All statistical analyses were performed using GraphPad Prism 8 software (GraphPad software, San Diego, CA). Significance was evaluated using two tailed unpaired t tests or non-parametric Mann-Whitney U tests where appropriate. Mouse experiments were done with 8 mice per group at each time point. Using a group size of 8 animals with a standard deviation of 35%, we are able to show a difference of >50% between two groups with a power of 80%. Results with a P-value of less than 0.05 were considered significant. ns: not significant.

## Supporting information

S1 Fig *DNMT3B* knockout and control BEAS-2B bronchial epithelial cells. (TIF)

S2 Fig Overexpression of Dnmt3b in bronchial epithelial cells does not influence *Pseudomonas aeruginosa* induced chemokine production *in vitro*. (TIF)

S3 Fig Heating does not eliminate flagellin activity towards BEAS-2B cells. (TIF)

S4 Fig Bronchial brushes are highly enriched for bronchial epithelial cells. (TIF)

S5 Fig Bronchial epithelial deficiency of Dnmt3b does not influence lung pathology or cytokine levels during pneumonia caused by wild-type *P*. *aeruginosa*. (TIF)

S6 Fig Bronchial epithelial deficiency of Dnmt3b does not influence lung pathology or cytokine levels during pneumonia caused by flagellin-deficient *P*. *aeruginosa*. (TIF)

S7 Fig Bronchial epithelial deficiency of Dnmt3b does not influence the host response during pneumonia caused by *Klebsiella pneumoniae*. (TIF)

S8 Fig Type II alveolar epithelial deficiency of Dnmt3b does not influence cytokine levels during pneumonia caused by *P*. *aeruginosa*. (TIF)

S9 Fig Gating strategy for neutrophils in BALF. (TIF)

S1 Table Primers used for RT-qPCR in this study. (DOCX)

S2 Table Primers used for MeDIP and ChIP in this study. (DOCX)
