# Supplementary figures and images for: Bronchial epithelial DNA methyltransferase 3b dampens pulmonary immune responses during Pseudomonas aeruginosa infection
Source: PLoS Pathog. 2021 Apr 1;17(4):e1009491. doi: 10.1371/journal.ppat.1009491 (PMC8043394; doi:10.1371/journal.ppat.1009491)

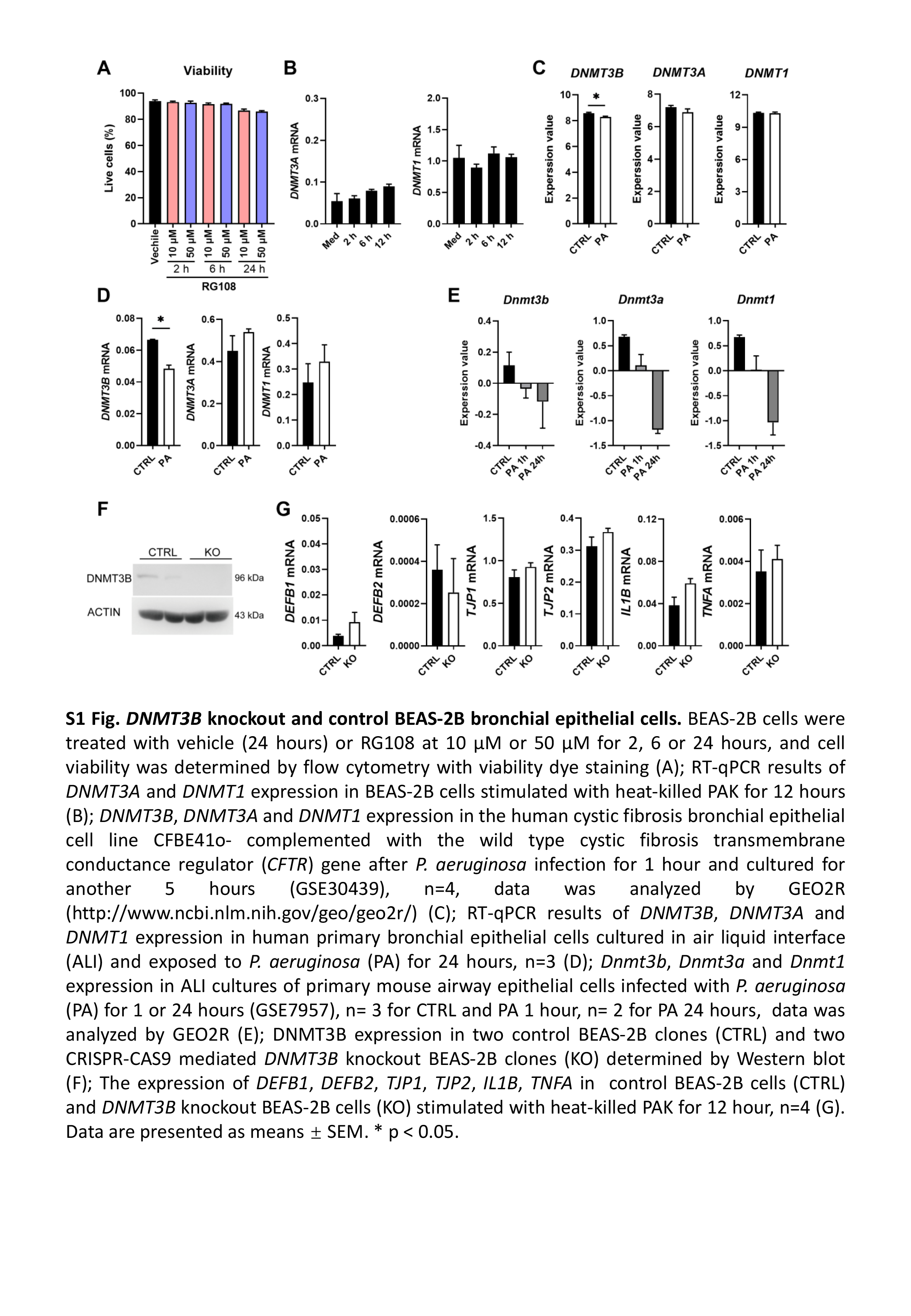

Supplement: S1 Fig — (TIF) [file ppat.1009491.s001.tif]

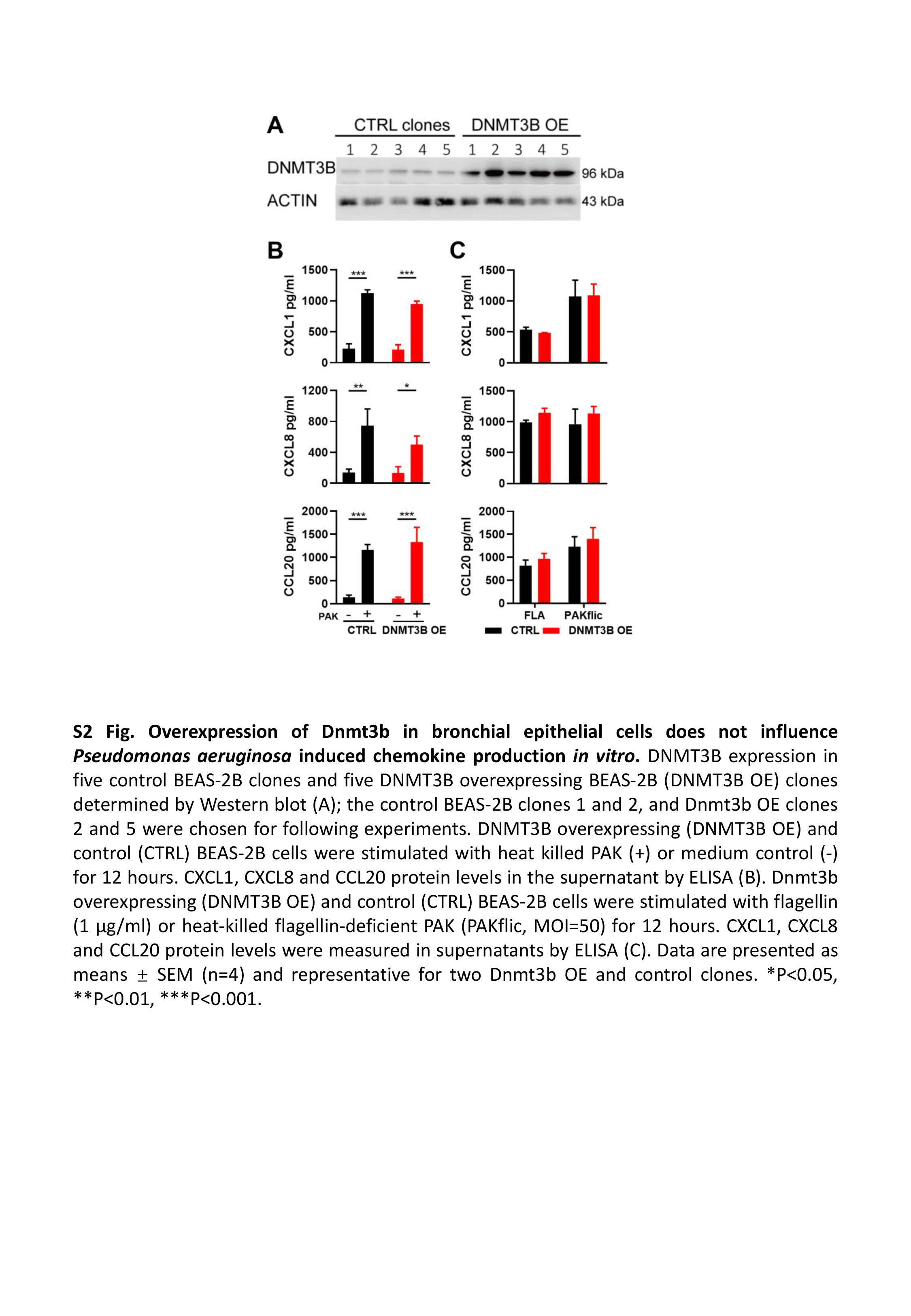

Supplement: S2 Fig — (TIF) [file ppat.1009491.s002.tif]

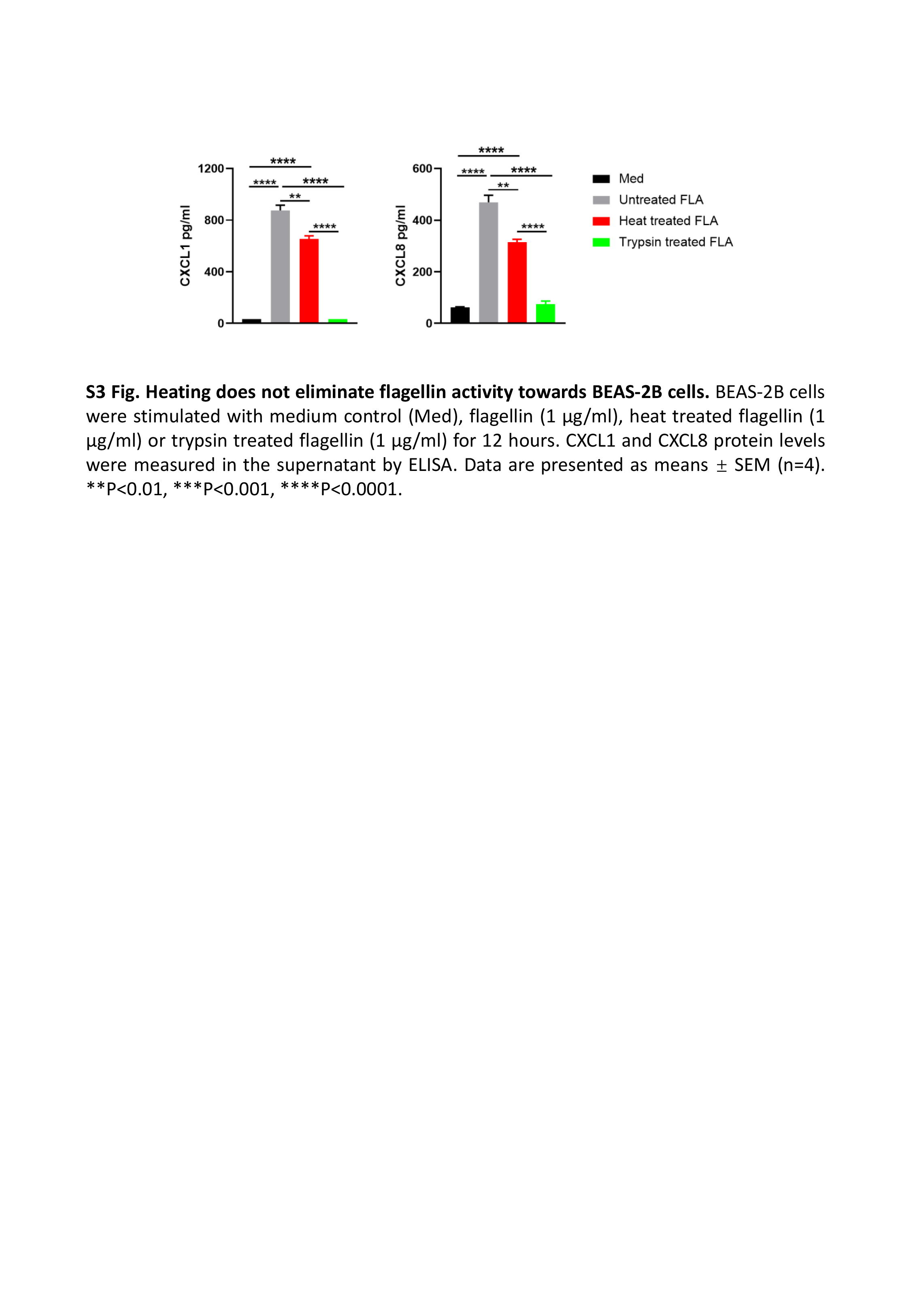

Supplement: S3 Fig — (TIF) [file ppat.1009491.s003.tif]

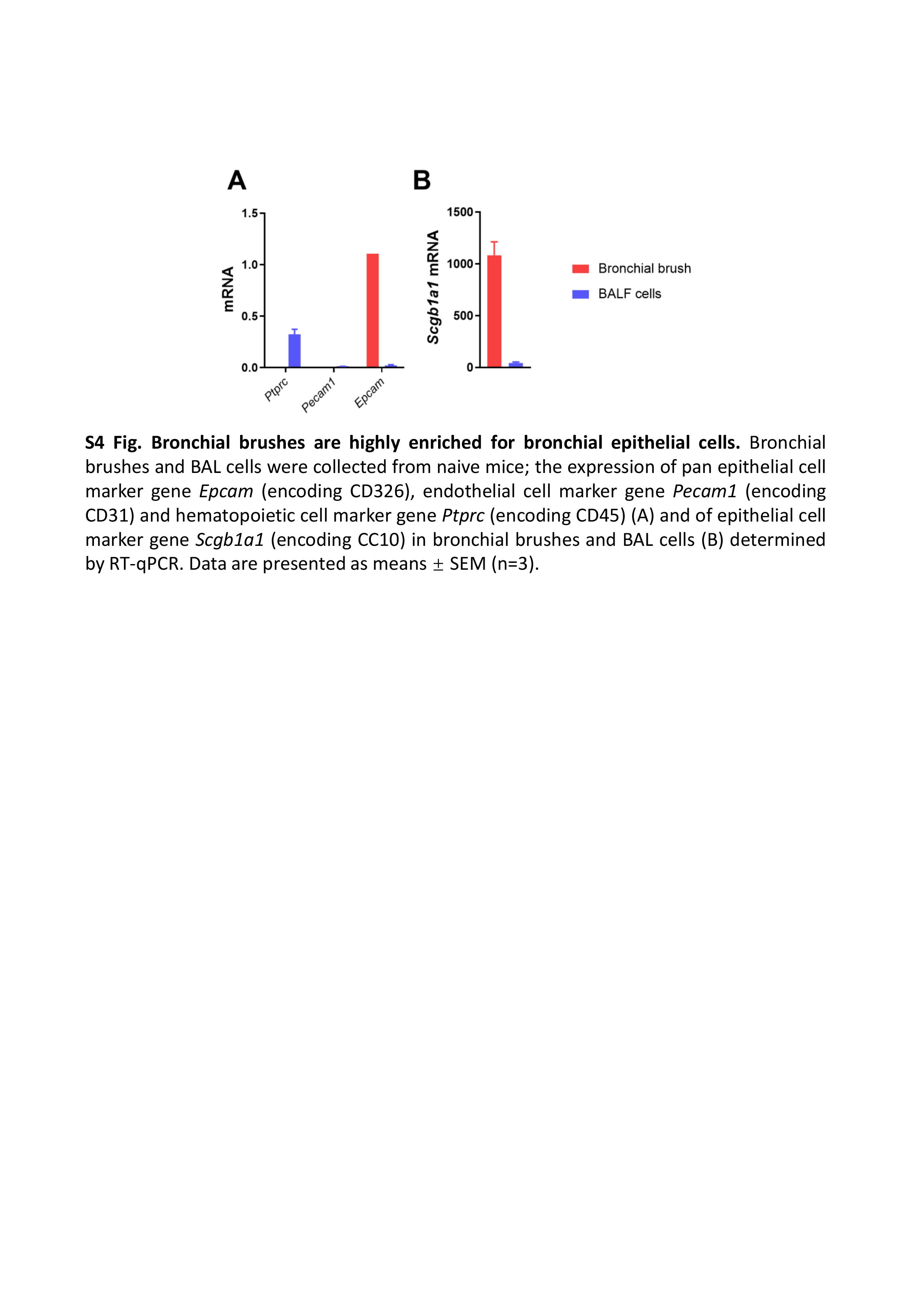

Supplement: S4 Fig — (TIF) [file ppat.1009491.s004.tif]

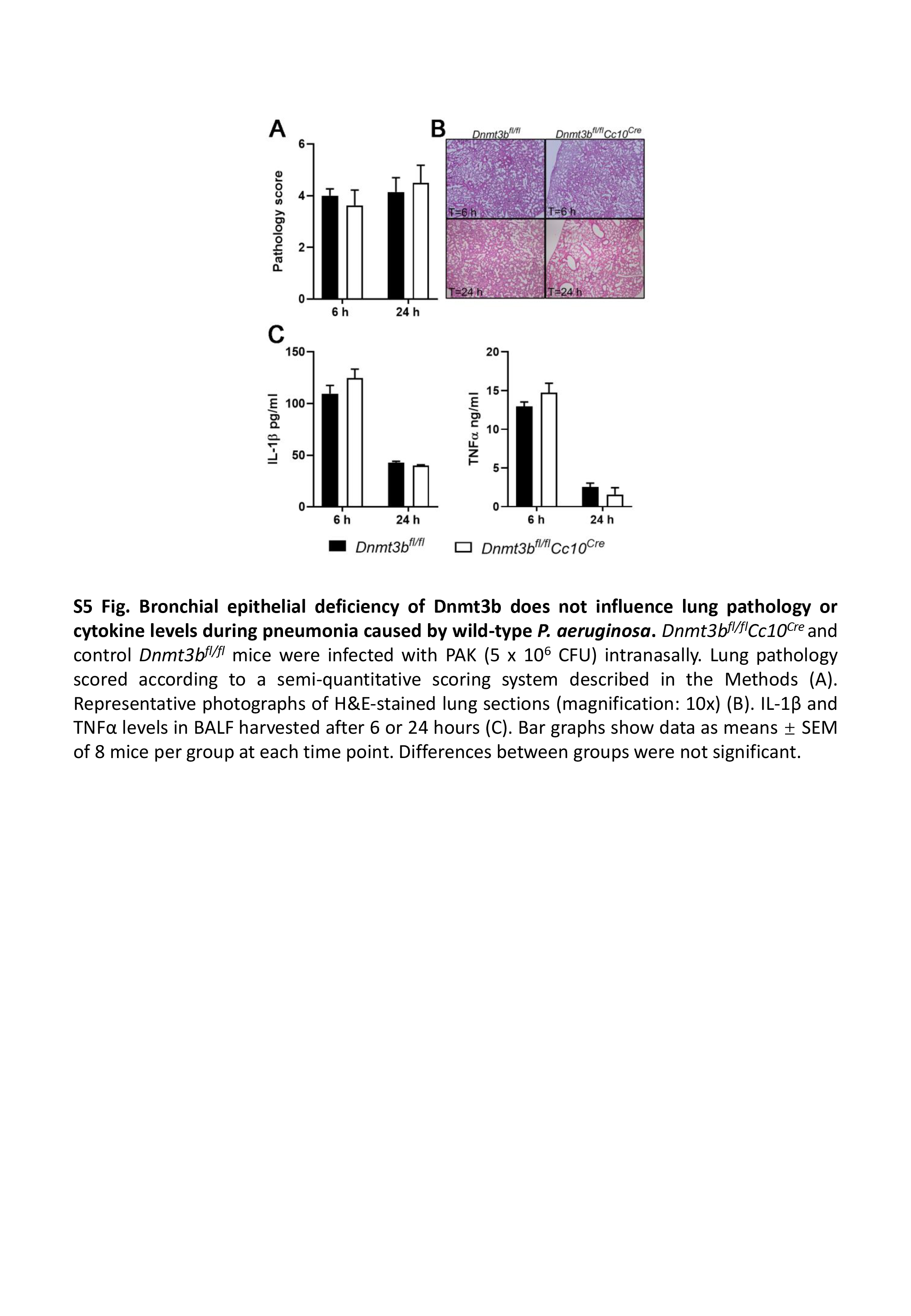

Supplement: S5 Fig — (TIF) [file ppat.1009491.s005.tif]

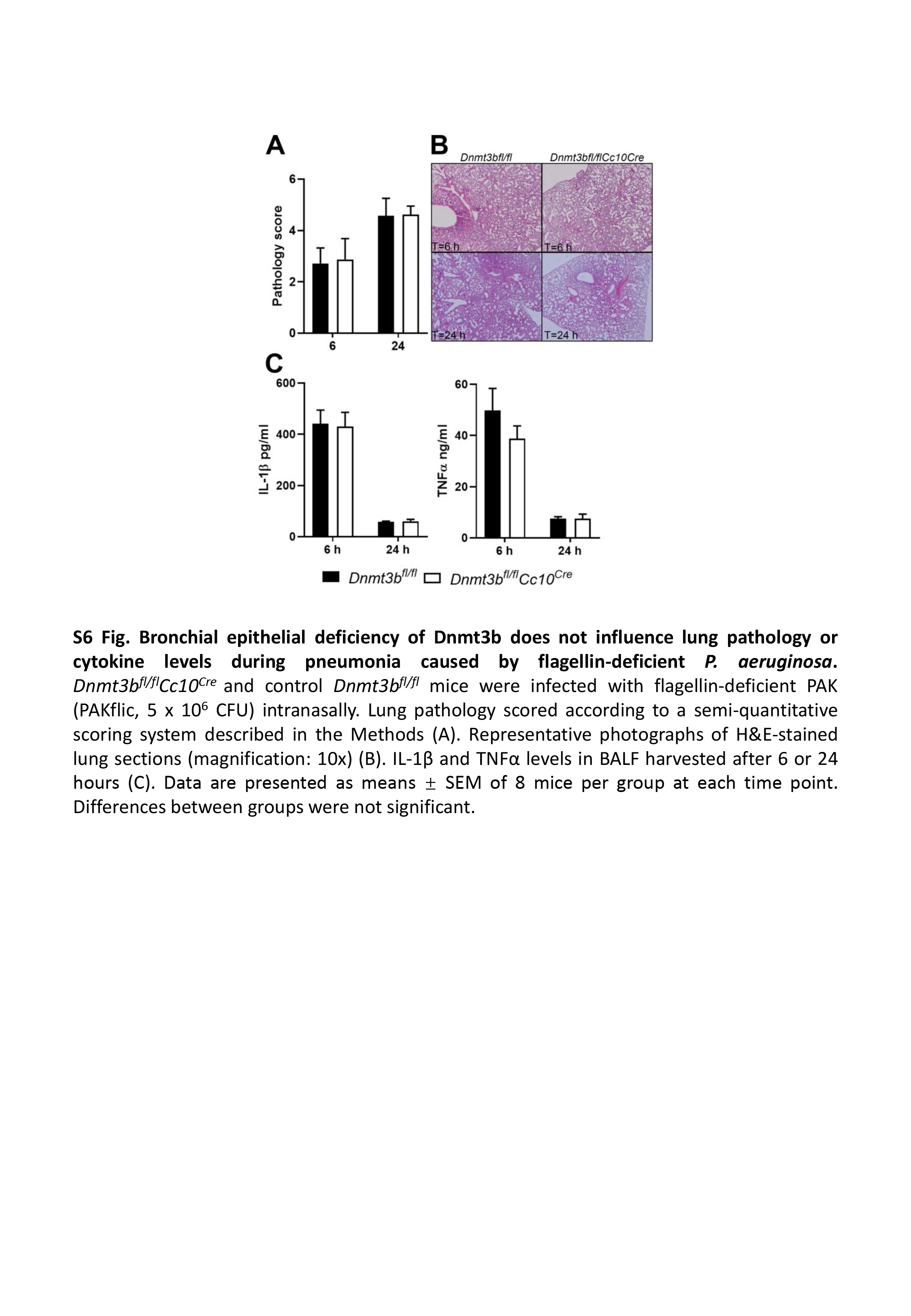

Supplement: S6 Fig — (TIF) [file ppat.1009491.s006.tif]

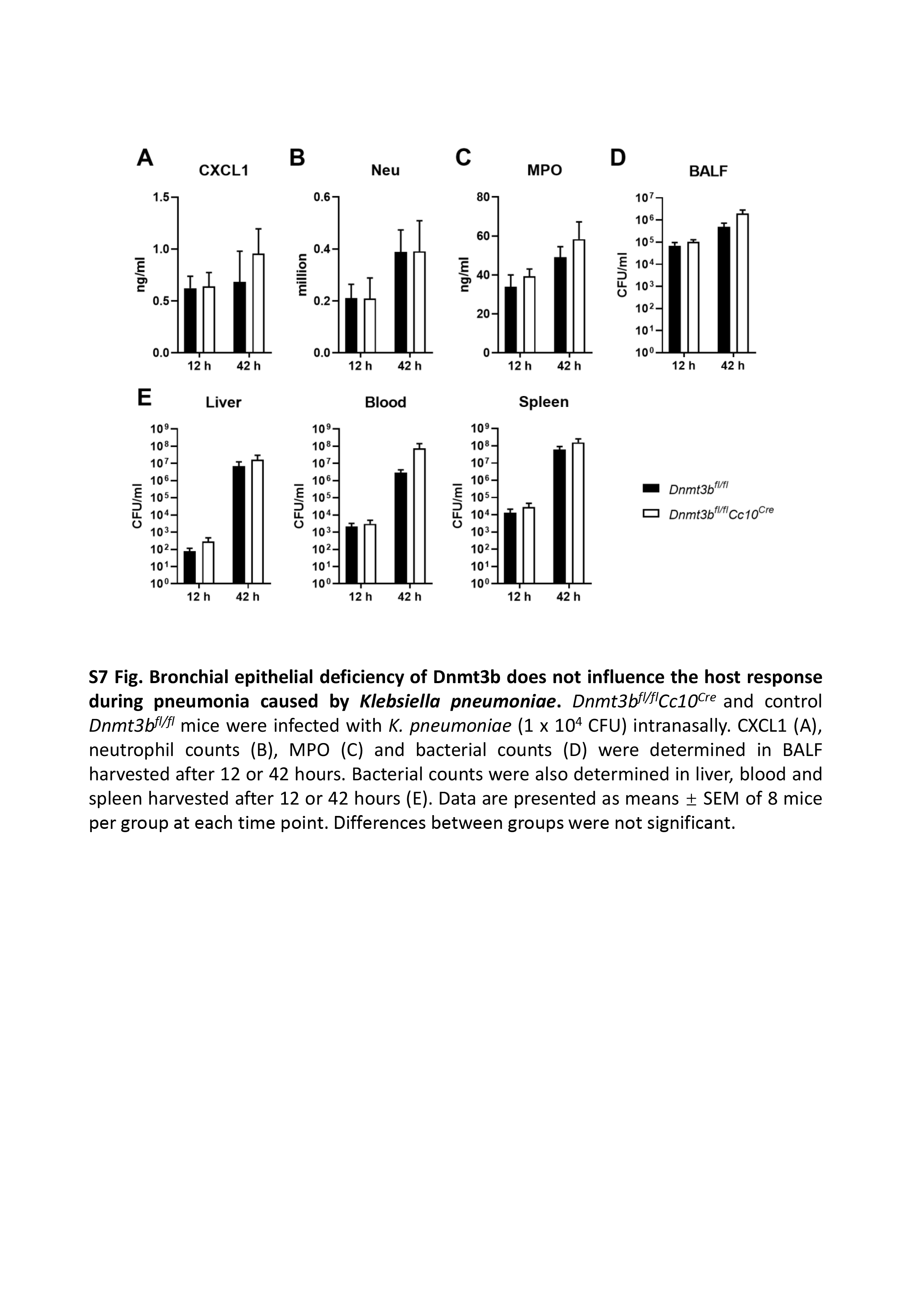

Supplement: S7 Fig — (TIF) [file ppat.1009491.s007.tif]

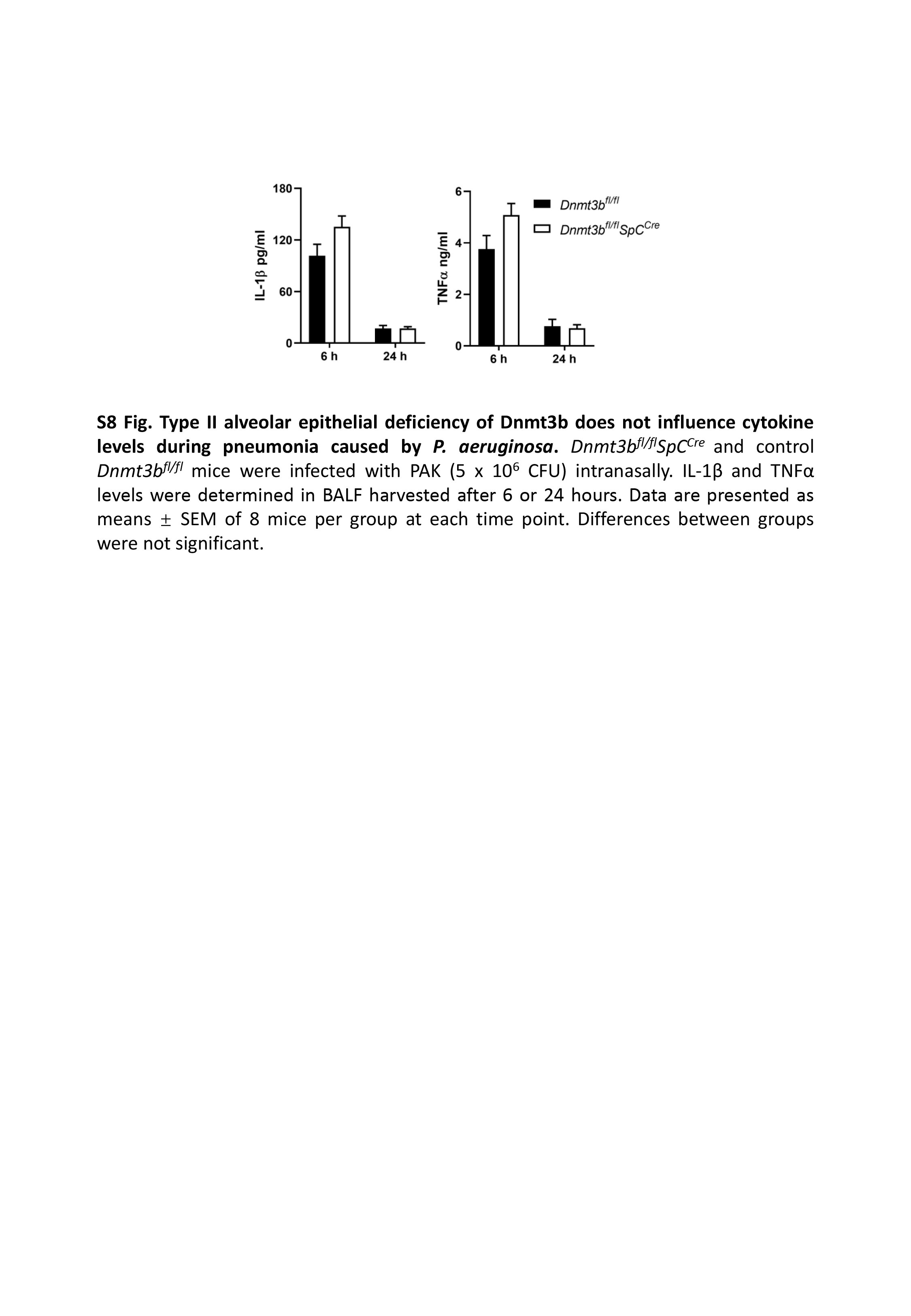

Supplement: S8 Fig — (TIF) [file ppat.1009491.s008.tif]

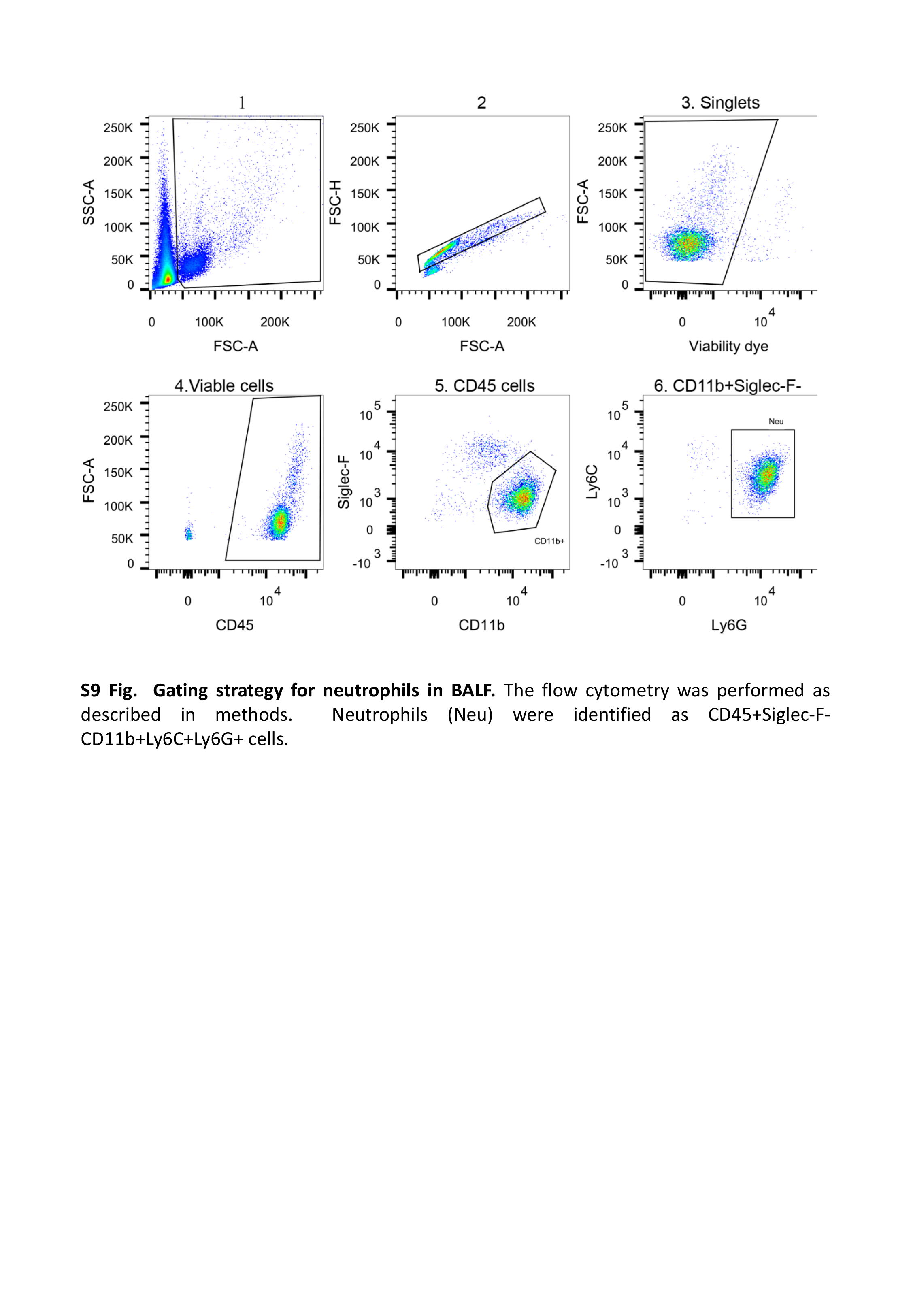

Supplement: S9 Fig — (TIF) [file ppat.1009491.s009.tif]
