# Supplementary material for: Bronchial epithelial DNA methyltransferase 3b dampens pulmonary immune responses during Pseudomonas aeruginosa infection
Source: PLoS Pathog. 2021 Apr 1;17(4):e1009491. doi: 10.1371/journal.ppat.1009491 (PMC8043394; doi:10.1371/journal.ppat.1009491)
Supplement: S1 Table — (DOCX) [file ppat.1009491.s010.docx]

**S1 Table, Primers used for RT-qPCR in this study.**

| **Species** | **Gene** | **Forward** | **Reverse** |
| --- | --- | --- | --- |
| **Human** | *DEFB1* | GATGGCCTCAGGTGGTAACT | AGATCGGGCAGGCAGAATAG |
|  | *DEFB2* | AGGCTTGATGTCCTCCCCAGA | GGCAGGTAACAGGATCGCCTA |
|  | *TJP1* | ACAGTGCCTAAAGCTATTCCTGTGA | TCGGGAATGGCTCCTTGAG |
|  | *TJP2* | CTTAGGGACAATAGCCCGCCC | TTTCATTACCAGCAACGGCAGA |
|  | *IL1B* | TGGCAATGAGGATGACTTGTTC | GCTGTAGTGGTGGTCGGAGATT |
|  | *TNFA* | CGAGTGACAAGCCTGTAGCC | CCTTGAAGAGGACCTGGGAGTA |
|  | *CXCL1* | GCATACTGCCTTGTTTAATGGT | CCAGTAAAGGTAGCCCTTGTTTC |
|  | *CXCL8* | AACCTTTCCACCCCAAATTTAT | AAAACTTCTCCACAACCCTCTG |
|  | *CCL20* | GATGTCAGTGCTGCTACTCCA | GCATTGATGTCACAGCCTTC |
|  | *DNMT1* | TACCAGGGAGAAGGACAGGG | ACACACCTCACAGACGCCAC |
|  | *DNMT3A* | AGGATAGCCAAGTTCAGCAAA | TGGACTGGGAAACCAAATACC |
|  | *DNMT3B* | CTGGAGGCTATCCGCACCC | GCCTGTCAAGTCCTGTGTGTA |
|  | *HPRT* | GGATTTGAAATTCCAGACAAGTTT | GCGATGTCAATAGGACTCCAG |
| **Mouse** | *Cxcl1* | CCACTGCACCCAAACCGAAG | TCCGTTACTTGGGGACACCT |
|  | *Cxcl5* | GCGGTTCCATCTCGCCATTC | TCCGTTGCGGCTATGACTGA |
|  | *Ccl20* | AGACAGATGGCCGATGAAGC | CTGCTTTGGATCAGCGCACA |
|  | *Ptprc* | TCCCCACTGTTTTGTTTACTCTTAC | ACACACGCCACATAAGCAAAG |
|  | *Pecam1* | AGTGGAAGTGTCCTCCCTTG | GAGCCTTCCGTTCTTAGGGT |
|  | *Epcam* | GTCCGAAGAACCGACAAGGA | TGATGGTCGTAGGGGCTTTC |
|  | *Scgb1a1* | CAGACACCAAAGCCTCCAAC | ATCCTGGGCAGATGTCCGAA |
|  | *Hprt* | AGTCAAGGGCATATCCAACA | CAGCCCCAAAATGGTTAAGGT |
