# Supplementary material for: Bronchial epithelial DNA methyltransferase 3b dampens pulmonary immune responses during Pseudomonas aeruginosa infection
Source: PLoS Pathog. 2021 Apr 1;17(4):e1009491. doi: 10.1371/journal.ppat.1009491 (PMC8043394; doi:10.1371/journal.ppat.1009491)
Supplement: S2 Table — (DOCX) [file ppat.1009491.s011.docx]

**S2 Table,** **Primers used for MeDIP and ChIP in this study.**

| **Species** | **DNA regions** | **Forward** | **Reverse** |
| --- | --- | --- | --- |
| **Human** | *CXCL1*_Promoter | AGTTACTCTGAAGGGCGAGGC | GCTCTGTGGCTCTCCGAGAT |
|  | *CXCL1*_Exon1 | CTCTTCCGCTCCTCTCACAG | CAGTGCCACTCGCAGGAG |
